# Src promotes castration-recurrent prostate cancer through androgen receptor-dependent canonical and non-canonical transcriptional signatures

**DOI:** 10.18632/oncotarget.14401

**Published:** 2016-12-31

**Authors:** Indranil Chattopadhyay, Jianmin Wang, Maochun Qin, Lingqiu Gao, Renae Holtz, Robert L. Vessella, Robert W. Leach, Irwin H. Gelman

**Affiliations:** ^1^ Department of Life Sciences, School of Basic and Applied Science, Central University of Tamil Nadu, Thiruvarur, Tamil Nadu, India; ^2^ Department of Bioinformatics, Roswell Park Cancer Institute, Buffalo, NY, USA; ^3^ Department of Cancer Genetics, Roswell Park Cancer Institute, Buffalo, NY, USA; ^4^ Department of Urology, University of Washington, Seattle, WA, USA; ^5^ Lewis-Sigler Institute for Integrative Genomics, Princeton, NJ, USA

**Keywords:** Src, androgen receptor, castration-recurrent prostate cancer, transcriptome, cistrome

## Abstract

Progression of prostate cancer (PC) to castration-recurrent growth (CRPC) remains dependent on sustained expression and transcriptional activity of the androgen receptor (AR). A major mechanism contributing to CRPC progression is through the direct phosphorylation and activation of AR by Src-family (SFK) and ACK1 tyrosine kinases. However, the AR-dependent transcriptional networks activated by Src during CRPC progression have not been elucidated. Here, we show that activated Src (Src527F) induces androgen-independent growth in human LNCaP cells, concomitant with its ability to induce proliferation/survival genes normally induced by dihydrotestosterone (DHT) in androgen-dependent LNCaP and VCaP cells. Src induces additional gene signatures unique to CRPC cell lines, LNCaP-C4-2 and CWR22Rv1, and to CRPC LuCaP35.1 xenografts. By comparing the Src-induced AR-cistrome and/or transcriptome in LNCaP to those in CRPC and LuCaP35.1 tumors, we identified an 11-gene Src-regulated CRPC signature consisting of AR-dependent, AR binding site (ARBS)-associated genes whose expression is altered by DHT in LNCaP[Src527F] but not in LNCaP cells. The differential expression of a subset (*DPP4*, *BCAT1*, *CNTNAP4*, *CDH3*) correlates with earlier PC metastasis onset and poorer survival, with the expression of *BCAT1* required for Src-induced androgen-independent proliferation. Lastly, Src enhances AR binding to non-canonical ARBS enriched for FOXO1, TOP2B and ZNF217 binding motifs; cooperative AR/TOP2B binding to a non-canonical ARBS was both Src- and DHT-sensitive and correlated with increased levels of Src-induced phosphotyrosyl-TOP2B. These data suggest that CRPC progression is facilitated via Src-induced sensitization of AR to intracrine androgen levels, resulting in the engagement of canonical and non-canonical ARBS-dependent gene signatures.

## INTRODUCTION

Prostate cancer (PC) is the second highest contributor to cancer-related deaths in U.S. men. Based on the critical role played by the androgen receptor (AR) as a transcriptional regulator of survival and proliferation genes in prostate cells, androgen-deprivation therapy (ADT), using AR antagonists or orchiectomy, has been highly successful in providing palliative benefit even in the setting of androgen-dependent (AD) metastatic disease. However, a significant portion of these men fail ADT after several years, resulting in castration-recurrent metastatic disease (mCRPC) that typically arises in the bone and lymph nodes, and that is largely driven by continued, often upregulated AR signaling [1, 2]. Indeed, higher levels of AR are thought to sensitize metastatic cells to low intracrine androgen levels expressed by tumor cells [3]. Second-generation AR antagonists, such as Enzalutamide (ENZ), have shown significant, yet non-durable benefit to some, but not all mCRPC populations [4, 5]. Interestingly, most cases of ENZ-resistant mCRPC exhibit either increased levels of wtAR, or the expression of ligand-independent AR mutants (e.g.- AR^F876L^) or splice variants (e.g.- AR-V7)[6, 7, 8, 9, 10, 11, 12]. Although some non-AR bypass mechanisms have been described [13], these data strongly suggest continued AR-dependence in mCRPC.

Several mechanisms have been described that facilitate AR activation in mCRPC following ADT failure. These include: i) AR mutations (primarily in the ligand-binding domain) that increase binding for non-androgen agonists [14], ii) AR stabilization [15], iii) induction of AR co-regulators [16] and iv) post-translational modification [14]. These changes are thought to facilitate AR-driven tumor progression in response to the post-castration expression of low levels tissue androgens [17]. Compared to AD-PC, mCRPC tissues exhibit increased protein tyrosine phosphorylation levels [18]. This correlates with increased activation levels of non-receptor tyrosine kinases, such as Src-family members (SFK) and ACK1 [19, 20, 21, 22, 23], presumably activated downstream of receptor tyrosine kinases, such as MET [24], KIT [25] and the EGFR family [26, 27], known to be activated in CRPC. Several SFK members, especially Fyn, and ACK1 are overexpressed in CRPC compared to primary PC tissues [2, 28], and these levels correlate with poorer prognosis [29]. Importantly, SFK and ACK1 can directly phosphorylate AR on Ty534 and Tyr267, respectively, thereby promoting CRPC growth *in vitro* and *in vivo* [30, 23, 31, 2]. Indeed, SFK or ACK1 antagonists suppress CRPC progression in mouse tumor models [32, 33, 34, 31, 35, 36]. Taken together, these data strongly suggest that SFK or ACK1 antagonists might potentiate the clinical effect of ENZ in mCRPC cases. However, the use of SFK inhibitors such as Dasatinib, Saracatinib, KXO1 or Bosutinib, have had mixed clinical results on disease-free progression as monotherapies [37, 38, 39, 40, 41] or in combination with docetaxel [42] or a VEGFR inhibitor [43]. Nonetheless, there is evidence that several of these agents reduce bone turnover markers in patients with pre-existing bone metastases [44, 45], suggesting that targeting SFK in combination AR antagonists such as ENZ may show clinical efficacy.

Here, we dissected the transcriptional programs by which activated Src (Src^527F^) could induce CRPC progression. Specifically, we performed transcriptome and AR cistrome analyses to determine how dihydrotestosterone (DHT) and/or Src^527F^ affected the expression of AR-dependent and –independent genes associated with CRPC progression. To this end the transcriptomes and cistromes were compared to those produced previously on human CRPC tumors [46], as well as those we produced on human AD (VCaP) and CRPC cell lines (CWR22Rv1 and LNCaP-C4-2), and on AD and CRPC human LuCaP35.1 xenografts. Our data indicate that in the absence of DHT, Src can trigger an extensive set of AR-dependent genes normally induced by DHT. However, we identified two other Src-driven gene groups that correlate with CRPC progression: i) Src-regulated genes normally not responsive to DHT, yet dependent on AR, and ii) differentially-expressed genes enriched in CRPC tumors and cell lines that can be regulated by Src in the absence of DHT. We identify an 11-gene Src-regulated CRPC driver signature, a subset of which correlates with earlier metastatic onset. Lastly, in addition to known androgen-response element (ARE) motifs, several Src-induced non-canonical AR binding sites were identified that share binding motifs for FOXO1, topoisomerase IIβ (TOP2B) and ZNF217. The upregulation of TOP2B and ZNF217, and the downregulation of FOXO1, correlated with earlier metastatic recurrence and poorer survival in CRPC patients. Taken together, these data strongly suggest that Src directs CRPC progression by activating canonical and non-canonical AR-dependent transcriptional programs. These results also suggest that progression to CRPC should be sensitive to combining AR antagonists with SFK/ACK1 kinase inhibitors.

## RESULTS

### Src induction of androgen independence requires AR

**Figure 1:**
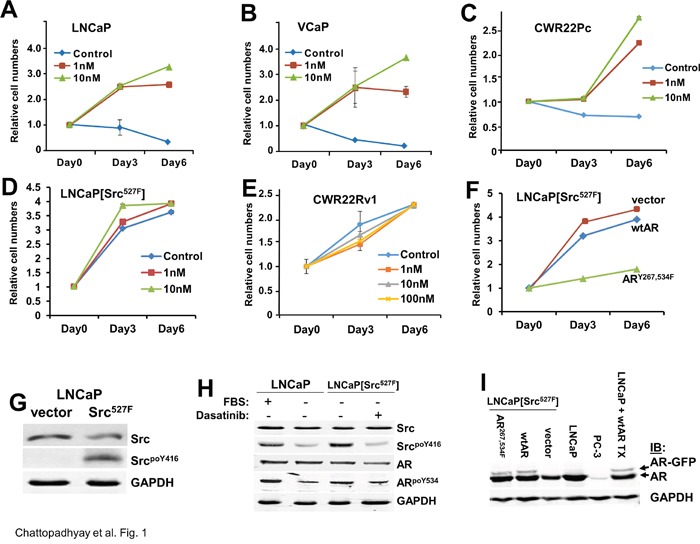
Src-induced androgen-independent growth requires AR tyrosine phosphorylation The proliferation of AD human PC cell lines, LNCaP **A**., VCaP **B**. and CWR22Pc **C**., LNCaP[Src527F] cells **D**., and the CRPC cell line, CWR22Rv1 **E**., was assessed (as described in Materials and Methods) in androgen-deprived conditions (Control) vs. DHT treatment (1, 10 or 100 nM). The proliferation of LNCaP[Src527F] cells transduced with empty vector, wt-AR or AR^Y267,534F^ was assessed in the presence of 1 nM DHT **F**. Error bars indicate mean ± SEM. Immunoblots showing total or active (Src^poY416^) in LNCaP or LNCaP[Src527F] cells **G**., the effect of serum (10% FBS) or Dasatinib (100 nM) on relative Src activation and AR^poY534^ levels LNCaP or LNCaP[Src527F] cells **H**., and the stable expression of ectopic AR-GFP alleles in LNCaP[Src527F] cells, compared to parental AR-positive LNCaP, AR-deficient PC-3, or transiently transfected LNCaP cells (“TX”) **I**. GAPDH blots were used as protein-loading controls. These blots are representative of at least three independent experiments each.

A hallmark of prostate cancer progression is its initial dependence on androgens for proliferation, followed by a transition to castration-recurrent growth in the absence of serum levels of androgens such as testosterone or DHT [47]. Previous data indicated that activation of Src, which is found in CRPC cell lines and human tumors, induces CR growth, likely through the direct phosphorylation and activation of AR [2]. Consistent with this, the addition of 1 or 10 nM DHT induced the *in vitro* proliferation of LNCaP, VCaP and CWR22Pc human PC cell lines, whereas incubation of these cells in androgen-free conditions (“control”) either caused growth-arrest or cell death (Figure 1A-1C). In contrast, expression of activated Src^527F^ induced androgen-independent growth (Figure 1D), consistent with a previous report [31]. Moreover, LNCaP[Src^527F^] cells, but not the CRPC line, CWR22Rv1 (Figure 1E), retained a small but significant responsiveness to 10 nM DHT. This is consistent with the notion that some CRPC cell lines and tumors, though able to grow in the absence of castrate androgen levels, remain androgen-responsive [47]. We also confirmed that LNCaP[Src^527F^] cells expressed high levels of autophosphorylated Src, Src^poY416^ (Figure 1G), previously shown to act as a surrogate marker for Src kinase activation levels [48].

A mechanism proposed previously for Src to activate androgen-independence was through the direct phosphorylation of AR at Y534 [30, 31, 32, 33]. Thus, we determined whether Src activation levels correlated with changes in AR^poY534^ levels. The addition of androgen-depleted FBS (charcoal-treated) to LNCaP cells grown in androgen deprived conditions resulted in the induction of Src activation as well as an increase in relative AR^poY534^ levels (Figure 1H). This agrees with previous data showing the ability of growth factors such as EGF to induce AR^poY534^ levels in LNCaP cells [31]. The high basal Src activation levels found in LNCaP[Src^527F^] cells could be blocked by the tyrosine kinase inhibitor, Dasatinib, and this correlated with a decrease in relative AR^poY534^ levels. Based on previously data showing that i) AR could also be activated through the direct phosphorylation on Y267 by the non-receptor ACK1 tyrosine kinase [23] and ii) that Src can activate ACK1 [49], we produced an AR-GFP construct containing Y→F mutations at residues Y267 and Y534, and then produced LNCaP[Src^527F^] cells that stably expressed wtAR or AR^Y267,534F^ (Figure 1I). In contrast to vector- or wtAR-expressing LNCaP[Src^527F^] cells, the ectopic expression of AR^Y267,534F^ blocked androgen-independent proliferation (Figure 1F), even though it is much less abundant than the endogenous AR (Figure 1I). This may be due to its ability to multimerize with endogenous AR, thereby inhibiting proliferative gene expression. Taken together, these data strongly suggest that Src can induce androgen-independent AR activation through AR tyrosine phosphorylation. Importantly, androgen-independent growth of LNCaP was AR-dependent because the shRNA-mediated depletion of AR (Supplementary Figure 1A) prevented proliferation of LNCaP[Src527F] cells in ADM conditions (Supplementary Figure 1B).

### Src induces genes normally DHT-regulated in androgen-dependent PC cell lines

**Figure 2:**
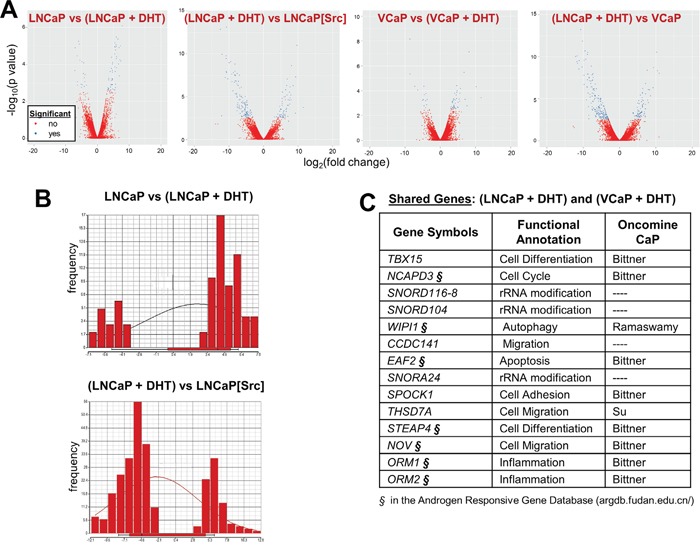
Gene expression trends induced by DHT or Src: Src downregulates whereas DHT upregulates **A**. Volcano plots comparing significantly up- (right arm) and downregulated (left arm) genes between PC cell lines +/- DHT. **B**. Comparison of differential gene expression levels with gene frequency, to assess the effects of DHT in LNCaP (top panel) or Src vs. DHT-regulated genes in LNCaP. **C**. Among the 15 most DHT-regulated genes shared in LNCaP and VCaP cells, several (§) were identified in the Androgen Responsive Gene Database (http://argdb.fudan.edu.cn/) or were shown to be differentially expressed in prostate cancer compared to control tissues in Oncomine studies (http://www.oncomine.org).

It is well accepted that androgens activate AR by inducing its nuclear translocation and subsequent function as a transcriptional regulator [16, 50]. Therefore, it is likely that Src can induce sufficient amounts of the AR transcriptional program normally induced by androgens in order to facilitate cell proliferation. In order to address this, we subjected LNCaP and LNCaP[Src^527F^] cells grown in the presence or absence of 10 nM DHT to AR cistrome analysis. We first performed comparative RNA-seq analysis, and included the androgen-responsive cells line, VCaP. Based on the work of Zhao et al [51], who showed that most androgen-regulated genes in LNCaP started to show expression changes at 6h, but peaked at 24h, we harvested RNAs for RNA-seq after 24h of DHT treatment (in androgen-depleted media), whereas AR-ChIP-seq analyses were performed on cells treated for 16h with DHT. All the library reads showed from 94.62 to 98.32% efficiency in calling correct bases, and additionally, the libraries yielded statistically similar Fragments Per Kilobase of transcript per Million (FPKM) mapped reads (Supplementary Figure 1C&1D), indicating comparable library sequencing/reading efficiencies. Interestingly, whereas DHT induced more gene upregulation in both LNCaP and VCaP, the expression of activated Src in the absence of DHT induced more gene downregulation compared to levels in DHT-treated LNCaP cells (Figure 2A and 2B). VCaP cells, which were derived from a CRPC metastatic lesion [52], also exhibited more gene downregulation in the absence of DHT when compared to levels in DHT-treated LNCaP cells (Figure 2A). We also were able to identify a significant number of genes induced by DHT in both LNCaP and VCaP (Figure 2C), whose DHT-induced expression was confirmed by qRT-PCR (Supplementary Figure 2A). A list of the most significantly expressed DHT-induced genes (Figure 2C) shows that many were identified previously in the Androgen Responsive Gene Database (argdb.fudan.edu.cn/) or differentially regulated in studies comparing prostate cancer to normal tissue (www.oncomine.org).

**Figure 3:**
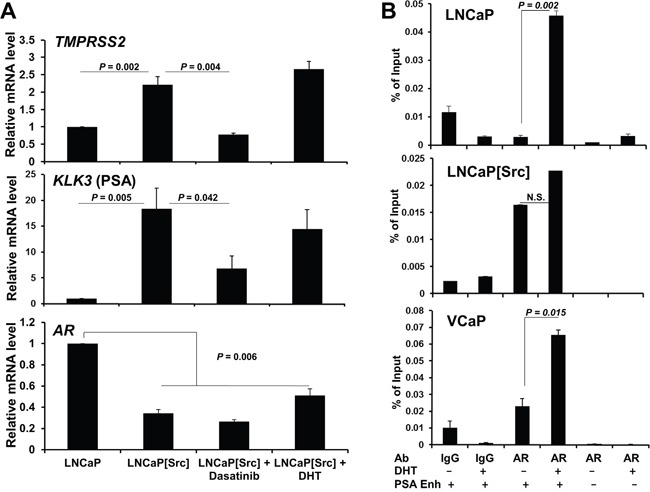
Src controls the expression of AR-dependent genes in a DHT-independent manner **A**. Measurement by qRT-PCR of *TMPRSS2, KLK3*(PSA) and *AR* in LNCaP or in LNCaP[Src527F] treated for 24h with vehicle (1% DMSO), Dasatinib (100 nM) or DHT (1 nM). **B**. ChIP assays on PC cells treated with vehicle or 10 nM DHT for 16h, using primers specific for the PSA enhancer (+) or a non-ARE PSA site (-), and AR-specific Ab vs. control IgG, as described in Materials and Methods. Error bars, mean +/- SEM from 3 independent experiments. N.S., not significant.

We next used qRT-PCR (as described in Materials and Methods, using primer sets described in Supplementary Table 1) to address whether a well-defined set of androgen-regulated genes, *TMPRSS2*, PSA (*KLK3*) and *AR*, could be regulated by the expression of activated Src. *TMPRSS2* and PSA expression, known to be induced by DHT in androgen-responsive PC cells [53, 54], was induced by activated Src, and this activation could be abrogated by Dasatinib treatment (Figure 3A). Moreover, 10 nM DHT treatment did not significantly alter *TMPRSS2* or PSA expression in LNCaP[Src^527F^] cells (Figure 3A), suggesting that Src-induces maximal AR activation in regards to these genes. In contrast, *AR* transcript levels, known to be downregulated in LNCaP by androgens [55], were downregulated by activated Src (Figure 3A). The finding that neither Dasatinib nor DHT reversed the downregulation of AR mRNA by Src strongly suggests that unlike the effects on *TMPRSS2* and PSA by Src, the effect on AR transcript levels is neither Src kinase nor AR-ligand dependent. The latter finding agrees with data showing that AR downregulation in this context involves the recruitment of the LSD1 demethylase to a novel AR binding site [56].

We then determined whether the ability of Src to regulate PSA transcription was due to increased AR binding to a well-described androgen-response element (ARE) in the PSA enhancer region [57]. In contrast to LNCaP and VCaP cells, where DHT induces AR binding to the PSA enhancer ARE, Src induced high levels of AR binding in the absence of DHT, and furthermore, DHT did not statistically enhance AR binding (Figure 3B). Indeed, Src-induced AR binding was identified at the *TMPRSS2* promoter and the *KLK3* enhancer, neither of which was enhanced by addition of DHT (Supplementary Figure 2B). These data are also consistent with the findings of Asim et al. [58] showing the requirement for Src kinase activity for the ligand-independent occupation by AR at the PSA enhancer ARE in the CRPC variant of LNCaP, C4-2 cells [59]. Src alone was able to induce *NKX3-1* expression (Supplementary Tables 6 and 9), and as well, was able to induce AR binding to the enhancer ARE in *NKX3-1* (Supplementary Figure 9A), whereas DHT suppressed *NKX3-1* expression and AR binding. In contrast, Src alone failed to induce AR binding to the *FKBP5* enhancer (Supplementary Figure 9B). Altogether, these findings suggest that Src can promote ligand-independent AR-mediated transcription, consistent with previously described AR activity in CRPC [60], although this was not universal for all DHT-regulated genes.

**Figure 4:**
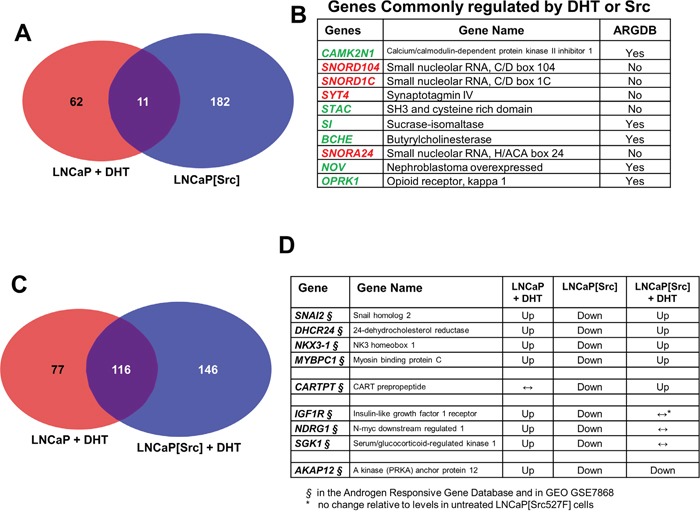
Genes regulated by Src in the presence or absence of DHT Transcriptome comparisons between LNCaP + DHT vs. LNCaP[Src^527F^] cells **A**., or between DHT-treated LNCaP vs. LNCaP[Src^527F^] cells **C**., with a differential expression threshold of log_2_^≥3^. **B**. The 10 most differentially expressed genes shared by DHT treatment or Src^527F^ expression in LNCaP cells (red: upregulated; green: downregulated). ARGDB: androgen responsive gene database (http://argdb.fudan.edu.cn/). **D**. Examples of genes whose DHT-induced expression (“Up”- or “Down”-regulation; ↔, no change) was altered in an opposite manner by Src. Note that all these genes were identified in the ARDGB or in GEO GSE7868 study of Wang et al. [75], which identified DHT-regulated genes in LNCaP cells.

A comparison of the transcriptomes from (LNCaP +/- DHT, or VCaP +/- DHT) vs. LNCaP[Src^527F^] cells was performed to identify Src-induced genes typically induced by DHT in LNCaP (Tables 1, Supplementary Table 2-10). This analysis identified 11 genes shared by the (LNCaP + DHT) vs. LNCaP[Src527F] cells, compared to 116 or 274 genes uniquely regulated by Src or DHT, respectively (Figure 4A, Table 1, Supplementary Table 8). Amongst the genes commonly regulated by DHT or Src (Figure 4B), four of the highest upregulated genes included those encoding three small nucleolar RNAs (*SNORD104*, *SNORD1C* and *SNORA24*) and Synaptotagmin IV (*SYT4*). Upregulation of several members of the so-called C/D-box small nucleolar RNA family have recently been shown to correlate with malignancy progression in prostate cancer [61]. Five of the most downregulated genes included those encoding calcium/calmodulin-dependent protein kinase II inhibitor-1 (CAMK2N1), STAC, Sucrase-isomaltase (SI), Butyrylcholinesterase (BCHE), NOV, and the Opioid receptor, kappa-1 (OPRK1). CAMK2N1, whose transcript levels are increased in primary PC yet downregulated in CRPC [62, 63] and whose downregulation predicts poor clinical outcome [64], encodes functions that suppress tumor invasiveness [65] and androgen-independent proliferation [66]. SI is downregulated in LNCaP cells overexpressing the AR co-activator, MED1 [67]. Battisti et al. [68] showed that serum BCHE levels decreased progressively in prostate cancer, and even more in patients with bone metastases, compared to control groups, and moreover, these decreased BCHE levels correlated with decreased biochemical recurrence-free survival [69]. The *NOV* gene, known to be transcriptionally downregulated by activated Src [70] and by a direct interaction between AR and the transcriptional repressor, EZH2, on the *NOV* promoter [71], may play a paradoxical role in promoting PC metastasis based on findings that its increased secretion by CRPC cells induces greater infiltration of pro-metastatic M2 macrophages [72]. The downregulation of *OPRK1*, known to be directed by AR [73], correlates with CRPC progression [74]. These data strengthen the notion that Src facilitates the up- and downregulation of normally DHT-regulated genes that drive CRPC progression.

**Table 1:** Genes Regulated by DHT, AR and/or Src

Experimental Condition	Control	DHT/AR-regulated Genes
LNCaP + DHT	LNCaP	*KLK2, IGF1R, NDRG1, SGK1, SLC5A4, SNAI2, TARP, HPGD, ELOVL7, ST6GALNAC1COL5A2, UGT2B17, UGT2B15, SI*
VCaP + DHT	VCaP	*KLK4, SLC2A5, TMPRSS2, DHCR24, AGR2VAV3, IGFBP3*
LNCaP[Src]	LNCaP + DHT	*PGR, PIGR, DPP4, LAMC3, HGMCS2,NKX3-1, SNAI2, TARP, ATAD2, ELOVL7, GHR,, HERC3, HOMER2, HPGD, JAG1, NDRG1, SGK1, SLC4A4, ADRA2A, ANXA1, CARTPT, HMGCS2, ID3, DHCR24, ETV1, IGF1R*
LNCaP[Src] + DHT	LNCaP[Src]	*SNAI2, CARTPT, DHCR24, NKX3-1*
LNCaP[Src]	LNCaP	*CD74, DPP4, PIP, SLC5A4,ETV1, GHR, JAG1, SI, SLC4A4, SLC22A3*

Sampling of highest up- (red) and down-regulated (green) genes differentially regulated (threshold of log_2_^≥3^) between control and experimental conditions.

When the transcriptomes of LNCaP and LNCaP[Src527F] cells treated with DHT were compared, a larger portion of genes (116) were regulated in both LNCaP and LNCaP[Src527F] cells (Figure 4C). This suggests that a set of genes regulated by Src might be super-regulated by DHT. Therefore, we analyzed datasets from the (LNCaP + DHT), LNCaP[Src527F] and (LNCaP[Src527F] + DHT) conditions to identify genes in which Src alters expression compared to DHT-treated LNCaP cells, or DHT alters Src’s ability to regulate expression in LNCaP[Src527F] cells. Four different gene expression themes were identified and examples of each are shown in Figure 4D. One gene group, containing *SNAI2*, *DHCR24*, *NKX3-1*, and *MYBPC1*, exhibited DHT-induced upregulation in LNCaP cells, downregulation in LNCaP[Src527F] cells, yet upregulation in LNCaP[Src527F] cells treated with DHT. A second group is typified by *CARTPT*, whose expression was unaltered by DHT in LNCaP, downregulated in LNCaP[Src527F] cells, yet upregulated in DHT-treated LNCaP[Src527F] cells. A third group, containing *IGF1R*, *NDRG1* and *SGK1*, was upregulated by DHT in LNCaP cells, downregulated by Src alone, yet unresponsive to DHT in LNCaP[Src527F] cells. Lastly, a fourth group, typified by *AKAP12*, showed DHT upregulation LNCaP, Src-induced downregulation, and further downregulation induced by DHT in LNCaP[Src527F] cells. When analyzed as a whole, these data describe a complex set of regulatory interactions between Src and AR. Furthermore, all the genes identified in this analysis are known to be androgen-regulated in LNCaP cells [75](GEO dataset GSE7868), strengthening the notion that Src (with or without DHT-induced super-effects) facilitates CRPC progression through AR activation.

In order to better understand the transcriptional networks regulated by either DHT or Src in LNCaP cells, we performed an Ingenuity Pathway Causal Networks analysis (http://www.ingenuity.com/products/ipa) using the differentially-regulated gene datasets obtained by RNA-seq, and then subtracted out networks shared by both DHT and Src (e.g.- cellular proliferation). As reported previously [76], DHT induced in LNCaP gene networks that regulate lipid metabolism and endocrine system development/function (Supplementary Figure 3). In contrast, Src (in the absence of DHT) induced gene networks that regulate cell survival, motility and amino acid metabolism (Supplementary Figure 4 & 5), which are hallmarks of malignant progression [1].

### Src-induced androgen-independent proliferation correlates with the regulation of CRPC-specific gene sets

LNCaP-C4-2 (“C4-2”) cells are metastatic variants of LNCaP cells derived in castrate mice [77]. In order to identify CR and DHT-regulated genes shared with either LNCaP or LNCaP[Src^527F^] cells, we performed RNA-seq analysis on C4-2 cells grown in the presence or absence of 10 nM DHT and then did pairwise comparisons of differentially expressed genes (Supplementary Tables 11-13). Specifically, we compared C4-2 to LNCaP, which would presumably identify C4-2-specific CR genes, or C4-2 +/- DHT treatment, which would identify androgen responsive genes in C4-2 cells. There was a strong overlap (>95%) between the C4-2-associated genes and those identified as a 73-gene signature in a previous analysis [78]. These groups were then compared to LNCaP vs. LNCaP[Src^527F^] cells (identifying genes induced by Src alone), or LNCaP+DHT vs. LNCaP[Src^527F^] (identifying Src-induced genes not also induced by DHT in LNCaP). Lastly, we identified genes shared by LNCaP[Src^527F^] and C4-2 cells (in the absence of DHT), presumably representing CR genes. This analysis showed that all of the genes identified in Figure 4B&4C as regulated by either DHT or Src in LNCaP cells could be induced in either untreated or DHT-treated C4-2 (Supplementary Table 2).

Interestingly, all the genes shown as regulated by DHT or Src in Figure 4B were identified as differentially expressed in untreated or DHT-treated C4-2 cells (Supplementary Table 2A [(LNCaP vs. LNCaP[Src^527F^]) & (C4-2 vs. C4-2+DHT)]; Supplementary Table 2B: [(LNCaP vs. LNCaP[Src]) & (LNCaP vs. C4-2)]), suggesting that these represent potential CR genes. Similarly, most of the Src-regulated genes described in Figure 4D were differentially expressed in DHT-treated C4-2 (Supplementary Table 2A: [(LNCaP+DHT vs. LNCaP[Src]) & (C4-2 vs. C4-2+DHT)]). This confirms that 95% of the genes most regulated by Src in LNCaP cells were differentially regulated in the CR variant of LNCaP cells, i.e.- C4-2, underlining the thesis that Src is a major driver of CR progression.

### Identification of an 11-gene Src-regulated CRPC driver signature

**Figure 5:**
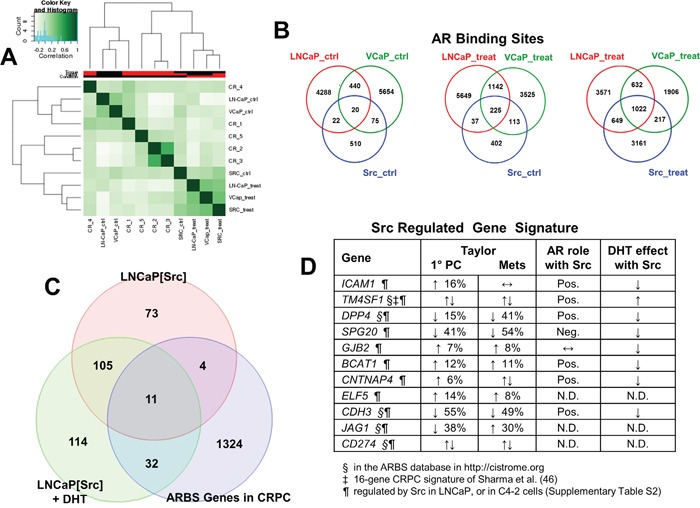
Src-regulated genes define a CRPC-driver signature **A**. Histogram showing concordance of ARBS between untreated (“ctrl”) vs. DHT-treated (“treat”) LNCaP, VCaP and LNCaP[Src^527F^] cells, or vs. CRPC lesions analyzed by Sharma et al. [46]. Venn diagrams showing frequency of ARBS overlap between untreated (“ctrl”) vs. DHT-treated (“treat”) LNCaP, VCaP and LNCaP[Src^527F^] cells **B**., or between untreated and DHT-treated LNCaP[Src^527F^] vs. CRPC lesions **C** and **D**. 11-gene Src-regulated CRPC signature. Previous identification of these genes in the ARBS database (http://cistrome.org)(§), the 16-gene CRPC signature of Sharma et al. [46](‡), or in our transcriptome analyses of Src-induced genes in LNCaP or genes regulated in the absence of DHT in C4-2 cells (¶) (Supplementary Table 2). Gene expression changes and the percent frequencies were assessed from primary PC lesions vs. metastases from the study of Taylor et al. [82], noting genes that were up- (↑) or down-regulated (↓), or that had no expression change (↔). Pos./Neg., indicates that the knockdown of AR by shRNA (Figure 6) either diminished or augmented gene induction by Src, respectively. N.D., not done.

If the Src-induced transcriptome program for androgen-independence correlates highly with known AR-regulated genes, then it is likely that Src also induces a concomitant increase in AR binding sites thought to be drivers of the AR regulatory cistrome in CRPC. We first compared the transcriptomes of the LNCaP, VCaP and LNCaP[Src^527F^] cells, plus or minus DHT (Figure 5A, “treat” or “ctrl”, respectively) with those published by Sharma et al. [46] involving five cases of CRPC metastatic lesions (GEO dataset GSE28219). As expected, this analysis showed strong correlation between untreated LNCaP and VCaP, and between untreated LNCaP[Src^527F^] and DHT-treated LNCaP and VCaP. With the exception of CRPC cases 2 and 3, which were highly similar in their transcriptomes, there was a varying degree of correlation between the five CRPC cases, consistent with the notion that mCRPC contains heterogeneous genetic changes [79, 6]. However, whereas all the CRPC samples except for case 4 correlated poorly with DHT-treated LNCaP, there was a consistent correlation between the LNCaP[Src^527F^] (treated or untreated) and all the CRPC cases. This suggests that Src induces part of the transcriptome that drives and/or maintains CRPC.

In order to map the Src-induced AR cistrome, we performed AR ChIP-seq on LNCaP, VCaP and LNCaP[Src^527F^] cells +/- DHT treatment. Galaxy Suite software was used to identify and compare overlapping sequencing read peaks immunoprecipitated (IP) by an AR-specific antibody (Ab), as described in Materials and Methods. As expected, DHT treatment increased the number of AR binding sites (ARBS) in both LNCaP and LNCaP[Src^527F^] cells (Supplementary Figure 6). DHT treatment also greatly increased the number of shared ARBS between the three cell types (Figure 5B: 1022 sites in treated vs. 20 sites in untreated cells). It is noteworthy that DHT treatment induced the greatest increase in AR binding events in the LNCaP[Src^527F^] cells compared to the LNCaP or VCaP cells (LNCaP[Src^527F^] cells: 629 ARBS in untreated vs. 5049 in treated; LNCaP: 4770 ARBS in untreated vs. 5874 in treated; VCaP: 6189 ARBS in untreated vs. 3777 in treated). Consistent with the notion that Src decreases basal AR binding events in LNCaP cells, there were fewer ARBS/per chromosome in untreated LNCaP[Src^527F^] vs. LNCaP cells, yet after DHT treatment, the total number of ARBS/chromosome between the two cell types was similar (Supplementary Figure 6). However, DHT treatment did not alter significantly where AR bound in regards to gene-associated or intergenic regions in LNCaP cells (Supplementary Figure 7). In agreement with previous studies [80, 81], at least 75% of AR binding sites in LNCaP cells were in intronic or distal intergenic regions. In contrast, there was a significant decrease in LNCaP[Src^527F^] cells in intronic AR-binding sites, with a concomitant increase in promoter, 5′-UTR and gene-flanking binding sites. We also performed several GREAT analyses to compare the effects of DHT or Src on i) the number of genes associated with ARBS (within 50Kb), ii) the distance (in Kb) of ARBS flanking known gene transcriptional start sites (TSS), and iii) the absolute distance (in Kb) between ARBS and TSS (Supplementary Figure 8). These confirm that Src suppresses the number of gene-associated ARBS in the absence of DHT (Supplementary Figure 8A-8C). After DHT treatment, there is a similar rise in ARBS associated with 2 genes in LNCaP and LNCaP[Src^527F^] cells, but paradoxically, there is an almost 2-fold higher number of ARBS not associated with local genes in the LNCaP[Src^527F^] cells (Supplementary Figure 8A). Similarly, the number of ARBS that flank known gene TSS is comparable between DHT-treated LNCaP and LNCaP[Src^527F^] cells (Supplementary Figure 8B). One possibly interesting difference is that DHT-treated LNCaP[Src^527F^] cells have about two-thirds fewer ARBS within 5Kb of TSS, and a concomitant increase in more distal ARBS (50-500Kb from TSS) than in DHT-treated LNCaP cells (Supplementary Figure 8C).

**Figure 6:**
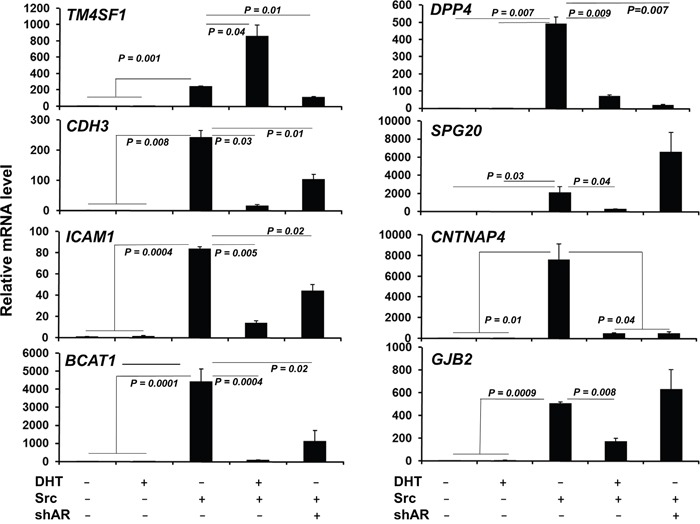
Most Src-regulated CRPC genes are controlled by AR but not by DHT qRT-PCR analysis of Src-regulated CRPC signature genes (Figure 5D) in LNCaP (-) or LNCaP[Src^527F^] cells (+) treated with DHT (10 nM) or vehicle for 24h, or in LNCaP[Src^527F^] cells expressing shAR (Supplementary Figure 1A). Error bars indicate mean ± SEM.

We then compared the genes regulated in LNCaP[Src^527F^] cells (+/- DHT) with AR cistrome genes identified in CRPC tissues by Sharma et al. [46] (Figure 5C). 11 differentially expressed genes were shared by LNCaP[Src^527F^] cells and CRPC tissues (Figure 5D), including three genes (*TM4SF1, DPP4* and *CDH3*) known to be androgen-regulated in LNCaP cells, five genes (*TM4SF1*, *DPP4*, *CDH3*, *JAG1* and *CD274*) previously identified in the AR binding database (http://cistrome.org), and one gene, *TM4SF1*, which was part of a 16-gene CRPC gene signature defined previously [46]. Most significantly, all the genes in our signature (Figure 5D) were also induced in LNCaP[Src^527F^] and C4-2 cells in the presence or absence of DHT (Supplementary Table 2), strengthening the notion that these may represent a CR signature of a Src-regulated AR cistrome. Interestingly, the genes in this signature were not induced by DHT in LNCaP cells (Figure 6). However, although they were induced significantly by Src in the absence of DHT, the Src-induced expression was either enhanced (*TM4SF1*) or suppressed by DHT (*DPP4*, *CDH3*, *SPG20*, *ICAM1*, *CNTNAP4*, *BCAT1* and *GJB2*), indicating that their expression remains AR-regulated. Indeed, with the exception of *GJB2*, the shRNA-mediated knockdown of AR (Supplementary Figure 1A) in LNCaP[Src^527F^] cells either decreased (*TM4SF1*, *DPP4*, *CDH3*, *ICAM1*, *CNTNAP4* and *BCAT1*) or increased (*SPG20*) transcript levels over those in untreated LNCaP[Src^527F^] cells (Figure 6). Taken together, these data suggest that Src regulation of CRPC genes remains AR-dependent (consistent with the fact that these genes were identified as containing ARBS in the CRPC tissues), yet is governed pleiotropically by AR ligand. One possible explanation of this complex interplay is shown in the MACS analysis of AR binding peaks for the *DPP4* gene (Supplementary Figure 9C). An AR binding peak (#2726; red circle), found in LNCaP cells, is enhanced after DHT treatment in LNCaP and in LNCaP[Src^527F^] cells, yet is absent in untreated LNCaP[Src^527F^] cells. Yet, *DPP4* expression is not affected by DHT treatment in LNCaP cells, but is upregulated in untreated LNCaP[Src^527F^] cells (Figure 6). Thus, the Src-regulated expression of *DPP4* cannot be explained by MACS peak #2726, but rather may be facilitated by a novel AR-binding peak (green circle) only found in LNCaP[Src^527F^] cells. In comparison, the DHT-inducible expression of *ADAM2*, which is not Src-regulated, is likely facilitated by a shared ARBS (Supplementary Figure 10).

**Figure 7:**
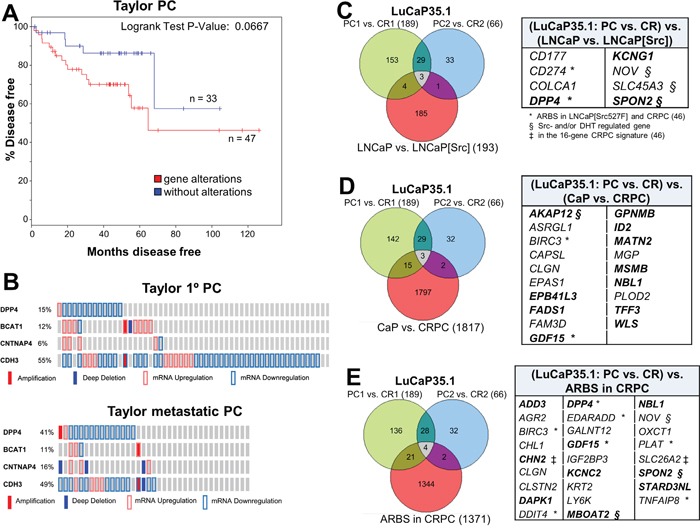
Src-regulated CRPC gene expression patterns correlate with earlier disease progression **A**. The differential expression of *DPP4*, *BCAT1*, *CNTNAP4* and *CDH3* in primary PC cases assessed in cBioPortal using the primary PC dataset of Taylor et al. [82], showed earlier onset of disease progression based on time to biochemical recurrence. **B**. The frequency of expression and copy number changes of *DPP4*, *BCAT1*, *CNTNAP4* and *CDH3* in samples from primary PC or metastases. Overlap of differentially-expressed genes (threshold of log_2_^≥3^) in CR vs. AD (PC) LuCaP35.1 xenografts compared to Src-regulated genes in LNCaP cells **C**., CR-associated genes in tumors analyzed by Sharma et al. [46] **D**., and CRPC ARBS **E**. Genes common to each group are shown in the panels on the right. Bold, differential expression correlates with CRPC metastases in Taylor et al. [82]. *, genes identified by ARBS analysis in LNCaP[Src527F] cells. §, Src- and/or DHT-regulated genes. ‡, identified in the 16-gene CRPC panel of Sharma et al. [46].

In order to address whether the altered expression of the Src-induced CRPC genes has relevance to clinical CRPC progression, we interrogated the dataset from mCRPC samples from Taylor et al. [82] using cBioPortal (http://www.cbioportal.org). The differential expression of the total 11-gene set (Figure 5D) correlated with a slight, but statistically non-significant, increase in earlier onset of metastatic disease (not shown). Thus, we attempted to identify trends in differential expression correlating with either primary PC or CRPC cases. For example, we excluded *ICAM1, TM4SF1* and *CD274* because clinical cases had no consistent trends (either up- or down-regulation) of these genes (Figure 5D). Of the remaining 8 genes, the differential expression of 4 genes, *DPP4*, *BCAT1*, *CNTNAP4* and *CDH3*, each showed a trend towards predicting worse survival, and in combination, this 4-gene set correlated with earlier onset of metastatic disease compared with cases without expression changes in these genes (Figure 7A, p=0.0667). The differential expression of the other 4 genes, *SPG20, GJB2, ELF5* or *JAG1*, showed no correlation with survival or metastatic recurrence, either individually or as a group. Interestingly, *DPP4* was predominantly downregulated in primary PC in the Taylor study (just under 15% of 85 cases), however in metastatic lesions, *DPP4* downregulation was found in just under 40% of cases, representing a 2.7-fold increased correlation (Figure 7B). Whereas there was little change in the low frequency of *BCAT1* upregulation or *CDH3* downregulation between primary PC and metastases, there was a small but significant increase in the frequency of *CNTNAP4* deep deletion in metastases. Importantly, all four genes showed similar expression trends (downregulation of *DPP4, CNTNAP4* and *CDH3*; upregulation of *BCAT1*) in multiple published datasets of primary PC vs. mCRPC found in Oncomine (http://www.oncomine.org) (Supplementary Figure 11). In their totality, these data demonstrate a strong correlation between this Src-regulated 4-gene set and CRPC progression.

We addressed whether *ICAM1, DPP4*, *BCAT1*, *CNTNAP4* or *CDH3* were required for Src-induced androgen-independent growth. LNCaP[Src527F] or CWR22Rv1 cells were stably transduced with representative shRNAs, and gene knockdowns were verified (Supplementary Figure 12A) in LNCaP[Src527F] cells. Aliquots of cells expressing either gene-specific shRNAs or empty vector (EV) were grown in androgen-free media (“-DHT”) or media supplemented with 1 nM DHT. *CNTNAP4* seemed to be required for proliferation irrespective of the androgen condition because its knockdown slowed the growth of LNCaP[Src^527F^] (Supplementary Figure 12B) or CWR22Rv1 (Supplementary Figure 12C). In contrast, *ICAM1* loss prevented proliferation irrespective of androgen condition in LNCaP[Src^527F^] cells only. Knockdown of *BCAT1*, however, significantly decreased androgen-independent proliferation in LNCaP[Src^527F^] or CWR22Rv1, whereas knockdown of *CDH3* or *DPP4* decreased proliferation similarly under androgen-free or –supplemented conditions. This suggests that *BCAT1* plays a specific role in Src-induced androgen-independent growth.

We then compared our transcriptome and AR cistrome analyses to the transcriptomes of LuCaP35.1, an AR-positive human xenograft system that starts as an AD growth yet which gives rise to CR lesions after castration [83]. RNA isolated from 3 independent AD and CR LuCaP35.1 lesions were analyzed by RNA-seq and then compared to genes i) regulated by Src in LNCaP[Src^527F^] cells (Figure 7C), ii) regulated in CRPC vs. primary PC lesions from the Sharma et al. group [46](Figure 7D), and iii) near AR binding sites in CRPC samples (Figure 7E). Interestingly, when the three CR LuCaP35.1 samples were compared to the AD samples, the genes most differentially upregulated in the CR lesions were almost all interferon-inducible (Supplementary Figure 13A), possibly reflecting increased NFkB-regulated survival pathways in CR cells in response to recruited, pro-metastatic inflammatory cells [84]. When the CR-specific LuCaP35.1 differentially-expressed genes were compared to those induced by Src in LNCaP (Figure 7C, Supplementary Figure 13B), CRPC-associated differentially-expressed genes (Figure 7D, Supplementary Figure 13C) or ARBS genes (Figure 7E, Supplementary Figure 13D) in Sharma et al. [46], there were only small numbers of overlapping genes (8, 20 and 27, respectively). However, these genes showed strong overlap with those we identified as part of the Src-driven CRPC signature or those identified as part of the putative 16-gene CRPC driver signature by Sharma et al. [46], with the strongest correlations associated with Src-regulated genes in LNCaP (Figure 7C; 62.5%) and ARBS genes in CRPC (Figure 7E; 46%). These data, using an independent set of human CRPC lesions, strengthen the thesis that Src is a major driver of the gene expression program that induces CRPC progression.

### Src broadens the non-canonical ARBS repertoire in CRPC

**Figure 8:**
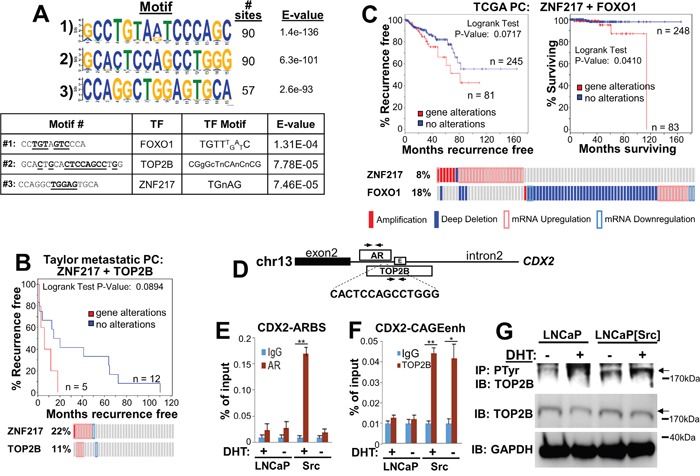
Src induces non-canonical ARBS that likely interact with CRPC-promoting transcription factors **A**. *Top panel-* The three most frequent non-canonical ARBS motifs and their prevalence in the LNCaP[Src^527F^] AR cistrome analysis. *Bottom panel-* identification of transcription factor binding site motifs (underlined) within the non-canonical Src-induced ARBS. **B**. Correlation between *ZNF217* and *TOP2B* differential expression and earlier metastatic recurrence from the study of Taylor et al. [82]. **C**. Correlation between *ZNF217* and *FOXO1* differential expression and earlier metastatic recurrence (left panel) and poorer survival (right panel) using PC samples (n = 326) from TCGA (http://cancergenome.nih.gov/). **D**. The *CDX2* Intron 2 contains an AR MACS peak (#3585) from our AR-ChIP-seq analysis of (LNCaP[Src^527F^] + DHT) cells, encoding the sequence, 5′-CACTCCAGCCTGGG, which is homologous to the non-canonical #2 motif described in panel A. This region also encodes an overlapping TOP2B MACS peak from estradiol-treated MCF-7 (GSE66753), as well as a CAGE enhancer (enh). Arrows, PCR primer sets. ChIP analysis of AR binding to the *CDX2-*ARBS **E**. or of TOP2B binding to the CDX2-CAGEenh **F**. from chromatin prepared from vehicle (-) or DHT (+) treated LNCaP or LNCaP[Src^527F^] (“Src”) cells. *, p<0.02; **, p<0.001. **G**. Lysates (1 mg protein/lane) from vehicle (-) or DHT (+) treated LNCaP or LNCaP[Src^527F^] cells incubated overnight with 4G10(anti-PTyr)-beads were analyzed by IB for TOP2B (arrow). Lysates (20 μg protein/lane) were analyzed by IB for TOP2B or GAPDH. Molecular weight markers are shown at right.

In addition to facilitating the activation of AR-regulated genes, Src also might facilitate CRPC progression by causing AR to bind to non-canonical ARE sites, presumably through novel functions gained by the direct phosphorylation of AR by Src. To analyze this, we identified ARBS in LNCaP[Src^527F^] cells that conformed to known AREs, and as well, novel non-canonical motifs. Using the “full” (AGRACAnnnTGTYCT) or “half” (AGAACA or TGTYCT) ARE consensus motifs from JASPAR (http://jaspar.genereg.net/), no matches were found for full AREs, whereas 262 peaks contained the AGAACA consensus and 265 peaks contained the TGTTCT consensus. In contrast, the three most non-canonical motifs, found in 57 to 90 AR-binding peaks also contained binding consensus sites for FOXO1, topoisomerase IIβ (TOP2B) and ZNF217 (Figure 8A), suggesting that Src-activated AR might either form complexes with these factors or compete for their binding. Indeed, the differential expression of these three factors has been reported to contribute to prostate cancer progression. For example, FOXO1 antagonizes AR activity [85] and its downregulation contributes to increased metastatic activity of prostate cancer cells [65]. Complexes containing AR and TOP2B induce double-stranded breaks resulting in gene fusion products, such as TMPRSS2-ERG, contribute to PC progression [86]. Moreover, Haffner et al. [86] showed that DHT-induced transcription of PSA/*KLK3* or *TMPRSS2* are facilitated by AR-TOP2B complexes binding to promoter and enhancer ARE sites. Lastly, ZNF217 upregulation is associated with increased proliferation in PC [87]. Importantly, the differential expression of these genes correlated with earlier disease onset or poorer survival in several PC studies in cBioPortal. Specifically, the upregulation of ZNF217 and TOP2B correlated with an earlier onset of CRPC (Figure 8B) in the study of Taylor et al. [82], and the combined upregulation of ZNF217 and downregulation of FOXO1 correlated with earlier CRPC onset and decreased survival in the provisional PC dataset from TCGA (http://cancergenome.nih.gov/) (Figure 8C). It is noteworthy that of the 18% of cases in the TCGA-PC databank (total = 327 cases) with *FOXO1* alterations, 78% were related to homozygous deletions of *FOXO1*; of the 8% of cases with *ZNF217* alterations, 21% exhibited gene amplification and the rest (with one exception) exhibited transcriptional upregulation. These data strongly suggest that ZNF217 and TOP2B agonize the ability of Src-activated AR to facilitate transcriptional activity at non-canonical sites, whereas FOXO1 functions in this manner as an antagonist.

To address whether Src induces a functional interaction between AR and TOP2B, we compared AR MACS peaks from LNCaP[Src527F] cells with TOP2B MACS peaks from estradiol-treated MCF-7 cells (GEO study GSE66753), and identified overlapping AR and TOP2B ChIP peaks in two genes, *C7ORF63* and *CDX2*. In the latter case, the overlapping ChIP peaks (found in Intron2 of *CDX2*) flank a non-canonical ARBS motif (Figure 8D), CACTCCAGCCTGGG, similar to the one we identified in LNCaP[Src527F] cells only (Figure 8A; motif #2), as well as an enhancer identified by CAGE analysis [88]. Interestingly, *CDX2* is not identified in the ARDGB as a DHT-induced gene in LNCaP cells, nor is it identified in recent TOP2B ChIP-seq analyses [89, 90]. AR binding to this site is potentiated in DHT-treated LNCaP[Src527F] only (Figure 8E), whereas the binding of TOP2B to the same region is potentiated by Src but unaffected by DHT (Figure 8F). This suggests that Src might alter the transactivation function of TOP2B, possibly by direct phosphorylation. To address this, lysates from LNCaP or LNCaP[Src527F] cells grown in the presence or absence of 100 nM DHT for 6h were incubated with beads loaded with anti-phosphotyrosine (4G10) MAb, followed by immunoblotting of these IPs for TOP2B. Figure 8G shows an increase in the abundance of phosphotyrosyl-TOP2B in LNCaP[Src527F] cells irrespective of DHT, strongly suggesting that TOP2B is a substrate of Src or a Src-induced tyrosine kinase. Indeed, several potential tyrosine kinase sites on TOP2B are predicted using SCANSITE (http://scansite.mit.edu/), and several phosphotyrosyl TOP2B peptides have been identified in multiple mass spectrometry studies described in PhosphositePlus (http://www.phosphosite.org/). Taken together, these data suggest that Src and DHT act to induce cooperative AR/TOP2B binding to the *CDX2* site.

## DISCUSSION

Much attention has been focused on the ability of CRPC lesions to continue to proliferate in the absence of serum androgen levels [47, 91]. The continued expression of wt-AR in CRPC [79, 6] and evidence of continued androgen-responsiveness [17] to low, intracrine levels of androgens [1, 92] clearly underlines the concept that CRPC progression depends on an adaptive, obligatory role for AR [93]. Indeed, resistance to the AR antagonist, Enzalutamide, still correlates with AR signaling, either by upregulation of wt-AR or the expression of AR mutants or splice variants [94], arguing for continued AR targeting even after resistance to AR antagonists [95]. However, the notion that CRPC pathology is simply the result of continued proliferation mechanisms falls short because these lesions, invariably associated with peripheral metastases, most often retain low proliferative indices [47]. Yet, they gain parameters of metastatic growth such as increased invasiveness and novel survival crosstalk pathways with local microenvironmental cells, such as in the bone [96]. One attractive notion is that the activation of Src-family and ACK1 tyrosine kinases, likely induced by the amplification and/or activation of multiple growth factor receptors-mediated pathways in CRPC [6], can potentiate AR transcriptional activity by the direct phosphorylation of AR on Y267 and Y534 [2]. Moreover, activated SFK would likely be able to drive multiple AR-independent parameters of malignant progression found in CRPC, based on their known oncogenic functions [22, 97]. Given that Src- and ACK1-specific inhibitors can suppress CRPC growth in pre-clinical models [31, 98] these findings suggest that combining SFK- and ACK1 antagonists could potentiate current anti-AR therapies such as enzalutamide.

In the current study, we analyzed the AR transcriptome and cistrome that correlates with Src-driven androgen-independence in LNCaP cells, and then attempted to show that many of the same Src-regulated genes were also found in CRPC cell lines, such as C4-2, xenografts such as LuCaP35.1, or CRPC tumors [46]. We first showed that the ability of activated Src to induce androgen-independent LNCaP proliferation correlated with increased AR^poY534^ levels. Conversely, the inhibition of Src activity by the pan-tyrosine kinase inhibitor, Dasatinib, which ablated androgen-independence [31], decreased relative AR^poY534^ levels. Similarly, the ectopic expression of an AR^Y267,534F^ allele inhibited androgen-independent proliferation. Src also induces the androgen-independent expression of well-characterized AR-regulated genes, such as *TMPRSS2* and *KLK3* (PSA), and in the case of PSA, induces high levels of AR binding to the enhancer ARE in the absence of androgens. Taken together with the inability of DHT to increase activation of these genes or the binding of AR to the PSA enhancer in LNCaP[Src527F] cells, these data strongly suggested that Src induces androgen-independent proliferation through the activation the AR-specific transcriptome/cistrome.

Consistent with this notion, we identified a large group of DHT-regulated genes whose expression could be induced or repressed in a similar fashion by Src in the absence of DHT. Of the upregulated genes in this group, three encode so-called C/D or H/ACA box small nucleolar RNAs that modify rRNAs through post-translational 2’-O-methylation or pseudouridylation, respectively [99], which presumably regulate prostate cancer progression by modulating gene expression. A fourth upregulated gene, encoding Synaptotagmin IV, regulates secretory granules in neuroendocrine cells [100] and is part of a prostate cancer-specific neuroendocrine signature [101], suggesting a role in the neuroendocrine differentiation of prostate cancer [102]. Of the gene products that are downregulated in this list, OPRK1 likely controls neuroendocrine differentiation [74], NOV suppresses androgen-independent PC growth [71], CAMK2N1 suppresses growth factor-induced PI3K and MEK/ERK proliferative signaling in PC cell lines [64], STAC likely modulates proliferative signaling through its SH3 and cysteine-rich domains, butyrylcholinesterase (BCHE) likely suppresses cholinergic stimulation of PC proliferation [68], and loss-of-function mutations in sucrase-isomaltase (SI) have been associated with cancer-promoting metabolic reprogramming [103]. Importantly, in LNCaP cells, Src mainly affects gene expression specific to prostate epithelial cell pathways since there are few in common with those regulated by activated Src in MCF-10A breast epithelial cells [104].

The notion that Src activation of AR ablates androgen responsiveness is mitigated by our finding of a group of normally DHT-induced genes whose expression is suppressed by Src, and then upregulated by DHT in the presence of activated Src. For example, the Src-induced downregulation of *SNAI2, DHCR24, NKX3-1* and *MYBPC1*, which are normally up-regulated by DHT in LNCaP cells, and the concomitant ability of DHT to upregulate these genes in LNCaP[Src527F] cells, suggests that Src’s activation of AR is incomplete in regards to certain target genes, and that further activation can be facilitated by super-activation of AR by DHT or the upregulation of AR co-activators by DHT. Importantly, though, this finding indicates that Src induces androgen-hypersensitization rather than independence.

We confirmed the thesis that Src could induce CRPC progression genes by showing a strong overlap between the transcriptomes of C4-2 cells grown in the absence of DHT and of CR-lesions of LuCaP35.1. Network analysis of the androgen-independent Src-regulated transcriptome identified pathways controlling cell survival and motility, and amino acid metabolism, in contrast to the major lipid metabolism and endocrine development and function pathways induced by DHT in LNCaP cells. Taken together, these data strongly suggest that Src’s ability to induce AR-regulated genes plays a major role in CRPC progression, a finding consistent with a large corpus of data using human PC cell lines and CRPC tissues, mouse transgenic models (reviewed in [2]).

Our AR cistrome analyses indicate that the major effect of Src in the absence of DHT is to decrease the total number of ARBS, yet to engage many of those genes normally induced by DHT that facilitate proliferation and survival. Indeed, in the absence of DHT, Src induces AR binding to a larger percentage of gene-associated sites, such as within promoter and enhancer regions. We identify an 11-gene Src regulated CRPC signature based on its overlap with the AR transcriptome/cistrome from CRPC clinical samples described previously [46], and on its overlap with the CRPC transcriptomes of C4-2 cells and LuCaP35.1 tumors. This signature includes *ICAM1, TM4SF1, DPP4, SPG20, GJB2, BCAT1, CNTNAP4, ELF5, CDH3, JAG1* and *CD274*. All these genes were similarly regulated by Src in untreated LNCaP cells, or in untreated C4-2 cells or CR-LuCaP35.1 lesions, and one (*TM4SF1*) was part of the 16-gene CRPC signature described by Sharma et al. [46]. However, only five of these have known local ARBS (*TM4SF1, DPP4, CDH3, JAG1* and *CD274*). Consistent with this, none of the 8 signature genes we tested (*ICAM1, TM4SF1, DPP4, SPG20, GJB2, BCAT1, CNTNAP4, CDH3*) showed more than a 2.5-fold induction by DHT in LNCaP. Interestingly, all these genes exhibited a greater than 200-fold induction by Src, which, in most cases was dependent on AR; the exceptions were *SPG20*, where AR seemed to antagonize Src-induced expression, and *GJB2*, which seemed AR-independent. Moreover, in all the genes except *TM4SF1*, DHT treatment caused a reversal of Src’s effect; for *TM4SF1*, DHT caused an enhancement. Thus, these genes would likely not be identified in signatures based on androgen-inducibility, yet most are DHT-regulated in the context of activated Src. We propose that this 11-gene signature, represents those most likely to drive CRPC in the context of activated Src. Consistent with this notion, we identified a subset of this signature containing *DPP4*, *BCAT1*, *CNTNAP4* and *CDH3*, whose expression are either the same or increased in CRPC compared to primary PC, and whose gene alterations correlate with a more rapid onset of cancer. Interestingly, only *BCAT1* was required for Src-induced androgen-independent proliferation, suggesting that the other gene products might function in other CRPC-related biologies.

We also reasoned that Src might drive CRPC by altering the breadth of non-canonical ARBS. To this end, we identified non-ARE (whole or half) ARBS found in LNCaP[Src527F] but not in DHT-treated LNCaP cells. The three most prevalent of these non-canonical binding sites, which represent about 18-20% of all the ARBS, also contained potential binding motifs for FOXO1, topoisomerase IIβ (TOP2B) and ZNF217, all of which are known to contribute to AR-dependent CRPC growth [105, 106, 87]. *ZNF217* and *TOP2B* expression is upregulated in human CRPC whereas that of *FOXO1* is lost, mostly resulting from deep deletions. *ZNF217* upregulation correlates with the loss of several targeting miRNAs such as miR-24 and -22 [87], and the forced knockdown of *ZNF217* inhibits proliferation of LNCaP and DU145 [87]. Although ZNF217 has not been shown to bind to AR (http://www.immunobase.org), it does bind to the estrogen receptor (ER) [107, 108] and is appreciated to be a biomarker of ERα-positive breast cancers [109], suggesting that it may play a regulatory role in conjunction with nuclear receptors. In contrast, AR is known to bind directly to FOXO1 and TOP2B. Whereas androgen-activated AR blocks engagement by FOXO1 of promoter elements for apoptosis and cell cycle arrest genes [110], consistent with FOXO1’s role as a transcriptional repressor, AR-TOP2B complexes coordinately regulate gene expression (as shown by co-ChIP experiments) as well as androgen-induced double-strand breaks and gene rearrangements, correlating highly with *TMPRSS2-ERG* fusion-positive PC [86]. Consistent with this model, we demonstrate that AR and TOP2B cooperatively bind to a non-canonical ARBS motif in the *CDX2* gene only in LNCaP[Src527F]. The finding that DHT is required for AR binding to this site suggests that modification of AR by Src increases its affinity for some non-canonical sites, thereby facilitating its interaction with TOP2B. The finding that TOP2B only associates with the *CDX2* site, which also has been identified as an eRNA-encoding enhancer [88], suggests that Src also modifies TOP2B.

Importantly, our data indicate that these gene expression alterations correlate with a more rapid progression to metastasis and decreased overall survival. This suggests that Src drives CRPC through at least two AR-dependent mechanisms, one involving the induction of ARE-encoding genes and the other involving non-canonical ARBS. Moreover, regulation of the latter genes likely involves increased association of AR with TOP2B and ZNF217, and decreased association with FOXO1.

## MATERIALS AND METHODS

### Plasmid construction

The AR^Y267,534F^ mutant was produced by sequential PCR-mediated mutagenesis (QuikChange Site-Directed Mutagenesis Kit, Stratagene) on pEGFP-C1-AR (Addgene #28235; kind gift of Michael Mancini) using primers: AR^Y267^F, 5′-GGGATTGCATGTtCGCCCCACTTTTGGGAGTTCC-3′ and AR^Y267^R, 5′-GGA ACTCCCAAAAGTGGGGCGaACATGCAATCCC-3′, and AR^Y534^F, 5′-GGATAG CTACTCCGGACCTTtCGGGGACATGCGTTTGGAG-3′ and AR^Y534^R, 5′-CTCCAAACGCATGTC CCCGaAAGGTCCGGAGTAGCTATCC-3′. The resulting mutations were confirmed by Sanger sequencing using primers: AR-260S, 5′- AGGCGTTGGAGCAT CTGAGTCCAGG-3′ or AR-525S, 5′-CAGAGTGCCCTATCCCAGTCCCACTT-3′.

### Western blotting

RIPA lysates were analyzed as described previously [32] using primary antibodies for Src, Src^poY416^ (Cell Signaling Technology, Beverly, MA), GAPDH and AR (Santa Cruz Biotechnology, Santa Cruz, CA). Rabbit polyclonal anti-AR^poY534^ [30] was kindly provided by Yun Qiu, Univ. of Maryland.

### Cell culture and DHT treatment

LNCaP[Src527F] were produced by infecting LNCaP cells (ATCC CRL-1740) with packaged pBABE*puro*[Src527F] retrovirus, as described previously [111] and selecting in RPMI-1640 media supplemented with 10% FBS and puromycin (2 μg/ml). LNCaP[Src527F] cells stably expressing wt-AR-GFP or AR^Y267,524F^-GFP were selected in media containing G418 (400 μg/ml) after transfection. VCaP (ATCC CRL-2876) and PC-3 (ATCC CRL-1435) cells were grown in DMEM + 10% FBS, whereas LNCaP-C4-2 cells (kind gift of Leland Chung, Cedars-Sinai) were grown in RPMI-1640 + 10% FBS. For time-course analyses, cells were hormone-starved for 2 days in androgen-depleted media (ADM; Phenol Red-free RPMI or DMEM supplemented with 10% charcoal-stripped FBS (CSS; HyClone, Logan, UT)) plus either 1 nM DHT in 1% ethanol or vehicle alone at 37°C, 5% CO_2_ for either 24 h (RNAseq) or 16 h (ChIP seq). Three sets of AD and CR LuCaP35.1 snap-frozen tumor and RNA samples were kindly provided by R. Vessella, Univ. of Washington.

### Cell proliferation assay

LNCaP, LNCaP[Src527F] or VCaP cells were hormone starved for 2 days in ADM, then treated with 1% ethanol (Control) or DHT for 0, 3 or 6 d. Cell counts were determined by washing once with PBS, fixed at room temperature for 30 min with 4% glutaraldehyde, and after air drying, staining with 0.1% crystal violet (in 20% methanol) for 30 minutes, followed by a PBS wash and drying. Cells were lysed with 100μl of 33% acetic acid and absorbance was read at 630 nm.

### RNA-seq library preparation

Total RNA was isolated using Trizol (Invitrogen) as per the manufacturer’s instructions. RNA was quantified by absorbance at 260 and 280 nm using a NanoDrop ND-1000 (Wilmington, DE, USA). The quality of RNA was determined using an Agilent Bioanalyzer 2100 (Santa Clara, CA), requiring RIN (RNA Integrity Number) value of ≥6.5. rRNA depletion (cytoplasmic and mitochondrial) was performed on 200-400 ng of total RNA using the RiboZeroGold kit (Illumina, San Diego, CA) as per the manufacturer’s instructions. The entire rRNA-depleted fraction (ranging 4-22 ng) was used as input for library preparation using the ScriptSeq V2 RNA Seq library preparation kit (Illumina, San Diego, CA) as per the manufacturer’s instructions. Briefly, rRNA-depleted samples were chemically fragmented using the StarScript Reverse Transcriptase Buffer and the cDNA Synthesis Primer was annealed to the RNA. 5′ end-tagged cDNA (equivalent to the 3′ end of the original RNA) was produced by random-primed cDNA synthesis. This was followed by 3′-Terminal Tagging of the cDNA using the Terminal-Tagging Oligo to produce a template for cDNA extension. The resulting “di-tagged” cDNAs were purified using Qiagen MinElute PCR Purification Kit (Hilden, Germany), ligated to the NEBNext Illumina-compatible adaptor 5′-poGATCGGAAGAGCACACGTCTGAACTCCAGTC-U-ACACTCTTTCCCTACACGACGCTCTTCC GATC*T-3′ (*, phosphorothioate bond), and then indexed using the NEBNExt Universal primer, 5′-AATGATACGGCGACCACCGAGATCTACACTCTTTCCCTACACGACGCTCTTCCGATC*T-3′ plus 6-mer NEBNext indexed primer sets, 5′-CAAGCAGAAGACGGCATACGAGATCGTGATGTG ACTGGAGTTCAGACGTGTGCTCTTCCGATC*T-3′, to allow multiplexing. The size of all libraries was assessed using the 2100 Bioanalyzer and a high sensitivity DNA chip (Agilent Technologies, Inc, CA), and further quantified with the Qubit DNA Broad Range assay (Life Technologies, Carlsbad, CA).

### RNA sequencing data analysis

Sequencing reads were first mapped to the reference genome (human GRCh37/hg19) with TopHat v2.0.13 (https://ccb.jhu.edu/software/tophat/index.shtml), using Bowtie v1.1.0 (http://bowtie-bio.sourceforge.net/index.shtml) to break up and align the short reads. The resulting alignment files were analyzed by Cufflinks v2.2.0 (https://github.com/cole-trapnell-lab/cufflinks) to generate a transcriptome assembly for each condition. These assemblies were then merged together using the Cuffmerge utility v3 (http://www.broadinstitute.org/cancer/software/genepattern/modules/docs/Cuffmerge/3), such that the resulting assembly could provide a uniform basis for calculating gene and transcript expression under each condition. Relative gene expression levels, their statistical significances, and transcriptional vs. post-transcriptional regulation were calculated by analyzing the reads and merged assemblies with Cuffdiff (part of the Cufflinks package). Cuffdiff calculates expression in two or more samples and tests the statistical significance of each observed change in expression between them. The statistical model used to evaluate changes assumes that the number of reads produced by each transcript is proportional to its abundance but fluctuates because of technical variability during library preparation and sequencing associated with biological variability between replicates of the same experiment. We employed the unpaired t-test using GraphPad Prism version 5.0 for PC (GraphPad, San Diego, CA) to compare mRNA levels for each gene between DHT-, Src-or vehicle-treated cells. P <0.05 was regarded as the threshold value for statistical significance. Volcano plots and relative gene expression data were analyzed and graphed using Partek GenomicsSuite^®^ (http://www.partek.com/pgs).

### ChIP

Ten million cells were grown to 70–80% confluence in a 150 mm culture dish containing ADM for 48 h before stimulation with 10 nM DHT or vehicle 16 h. DNA-protein cross-linking was induced by incubating in 1% formaldehyde for 7 min at room temperature (RT), before quenching with a final concentration of 125 mM glycine. Cells were washed twice with 10 ml ice cold PBS supplemented with protease inhibitor cocktail (cOmplete^™^, Roche). Cells were harvested by scraping in PBS, followed by centrifugation at 800g at 4°C for 5 min and resuspending in 1 ml ChIP lysis buffer (50 mM Tris pH 8.1, 150 mM NaCl, 5 mM EDTA, 0.1% sodium dodecyl sulfate [SDS], 2% Nonidet P-40, 0.5% deoxycholate, 0.5 mM phenylmethanesulfonyl fluoride [PMSF] with 1X protease inhibitor cocktail). Nuclear lysates were divided into five 200 μl fractions, sonicated for 15 min (30 sec on, 30 sec rest) at maximum power in a Bioruptor sonication water bath (Diagenode) and pooled (total volume 1 ml). 100 μl of 10% Triton X-100 was added and insoluble debris was removed by centrifugation at 10,000 g for 10 min at 4°C. 100-200μL of supernatant was diluted with 2 ml of IP Dilution Buffer (0.01% SDS, 1.1% Triton X 100, 1.2 mM EDTA, 16.7 mM Tris-Cl pH 8.0, 167 mM NaCl) supplemented with 1 mM PMSF and 1X protease inhibitor cocktail. 50 μl was taken as total input control and the remainder was used for ChIP. For each ChIP reaction, the remaining sheared chromatin solution was incubated overnight at 4°C with gentle agitation with 20μL of pre-cleared Magna ChIP Protein A Magnetic Beads (Millipore; Cat#16-661) plus 10 μg of AR Ab (AR N-20, Cat. #SC-816X, Santa Cruz). This AR Ab was used in multiple AR-ChIP-seq studies [112, 75, 113]. Ab-bead complexes were washed three times with 1 ml of 0.5% BSA in PBS, resuspended in 100 μl of the same buffer, combined with the pre-cleared nuclear lysates and incubated overnight at 4°C with gentle agitation. The following day, the beads were washed five times with RIPA buffer (50 mM HEPES-KOH pH 7.6, 500 mM LiCl, 1 mM EDTA, 1% IGEPAL^®^ CA-630, 0.7% sodium deoxycholate), once with TE (10 mM TRIS, pH 7.4, 0.1 mM EDTA) plus 50 mM NaCl at 4°C and eluted in 200 μl elution buffer (50 mM Tris-HCL pH8, 10 mM EDTA, 1% SDS) for 15 min at 65°C with vortexing. Cross-links were reversed by overnight incubation at 65°C, and then RNA and proteins were degraded by adding 200 μl of TE and 8 μg of DNAse-free RNAse A (Ambion), incubating for 30 min at 37°C, followed by the addition of 80 μg Proteinase K (Invitrogen) and incubating at 55°C for 1 h. Genomic DNA was isolated using phenol:chloroform:isopropanol (25:24:1, Invitrogen), back-extracted with 200 μl of TE, precipitated with isopropanol, washed with 75% ethanol, air-dried and resuspended in 60 μl 10 mM Tris-HCl pH 8. ChIP-PCR was performed with 6 μl DNA using SYBRGreen dye kits (Applied Biosystems).

### ChIP-seq SOLEXA library preparation

Briefly, 10 ng of ChIP DNA was subjected to end-repair using T4 DNA polymerase, Klenow DNA polymerase and T4 polynucleotide kinase, before purification using the DNA Clean and Concentrator-5 kit (Zymo Research). Adenine overhangs were added using Klenow 5′-3′ exo-minus polymerase (New England Biolabs). Illumina Solexa sequencing adapters were ligated using T4 DNA ligase and amplified with 18 PCR cycles using Phusion DNA polymerase (Finnzymes) and Illumina Solexa sequencing primers 1.1 and 2.1. Libraries were size-selected by electrophoresis, excising the DNA smear between 200-300 bp on a Dark Reader non-UV transilluminator, purified using a Qiagen gel-extraction mini-elute kit and quantified using an Agilent Bioanalyser.

### Sequence-read analysis

Single-end 36 bp sequence reads were generated by the Illumina analysis pipeline versions 1.3.4 and 1.4.0. The two lanes of reads were combined for each sample, and aligned to the Human Reference Genome (assembly hg18, NCBI Build 36.1, March 2008) using Mapping and Assembly with Quality (MAQ; [114]). Next, they were filtered by alignment quality score, removing all reads with a MAQ score <20, and exact duplicate reads were removed such that no single read start position was represented more than once. Enriched regions of the genome were identified by comparing the ChIPed samples with input samples using two independent peak calling algorithms: MACS [115] and ChIPSeqMini [116], taking only those regions found by both algorithms. Sites found in the androgen stimulated and Src condition, but not the vehicle-treated condition, were analyzed further. Enriched regions of the genome were identified by comparing ChIP samples to input samples using SWEMBL peak caller version 3.2 (http://www.ebi.ac.uk/∼swilder/SWEMBL/), MACS and ChIPSeqMini. Only peaks that were present in >2 samples in each treatment group, and peaks that were identified by both MACS and ChIPSeqMini for cell lines, were used for further analyses. The Dreme software package [117], in the MEME suite, was used to search for enriched conserved motifs within AR binding peaks by comparing FASTA files of AR-ChIP-seq data to those using control ChIP Ab (representing random sequences).

### Analysis of ChIP peak regions

ChIP-seq enriched regions were identified using the Galaxy suite [118], based on overlap, subtraction, union and feature annotations. Gene annotation associated with ARBS was performed using Genome Regions Enrichment Annotations Tool (GREAT) analyses (http://bejerano.stanford.edu/great/public/html/splash.php). Transcription factor motifs were identified using CEAS, *de novo* motif searches using MEME [119] and Nested MICA [120] and position weight matrix searches using RSAT matrix-scan (http://rsat.ulb.ac.be/rsat/). MEME motifs were compared with random-generated sequences with the same base frequency. Motifs identified using *de novo* searches were aligned with known transcription factor position weight matrices using the motif alignment tool in the JASPAR database (http://jaspar.cgb.ki.se/). The optimal genomic distance between AR binding sites (peak boundaries) and androgen-regulated genes (gene boundaries) were defined using Gene Set Enrichment Analysis (GSEA: http://software.broadinstitute.org/gsea/index.jsp). Briefly, we generated gene sets by identifying all genes within 1, 2.5, 5, 25 and 100 Kb of AR binding sites, whereas control sets were generated by identifying genes with no adjacent AR binding sites within >50Kb. These gene sets were tested for enrichment of the 3319 androgen-regulated genes identified in our detailed expression profiling data using GSEA (using the ‘time course’ correlation).

### Quantitative real-time PCR

RNA (50 ng) was used for each reaction and the result was normalized by amplification of 18S RNA. Real-time quantitative PCRs were carried out in an ABI Prism 7900, using SYBRgreen PCR master mix (Applied Biosystems, Warrington, UK). Reactions were carried out in triplicate and with biological replicates. Primers are shown in Supplementary Table 1.

## CONCLUSIONS

This study identifies three major mechanisms by which Src promotes CRPC progression through the androgen-independent activation of AR-regulated genes targets. First, Src potentiates the ability of AR to regulate survival and proliferation genes normally controlled by DHT in AD-PC cells. Second, Src induces an 11-gene, AR-dependent signature that is enriched in CRPC cells and tissues, and that predicts poor clinical outcome. Third, Src induces CRPC-associated genes by increasing AR binding to non-canonical sites, enriched for FOXO1, TOP2B and ZNF217 binding motifs. The differential expression of Src-regulated CRPC signature genes and of *FOXO1*, *TOP2B* and *ZNF217* correlates with earlier metastatic onset and poorer clinical outcomes in PC patients, underlying the notion that Src is a critical driver of AR-dependent CRPC progression.

## SUPPLEMENTARY FIGURES AND TABLES



























## Abbreviations

AD: androgen-dependent
ADM: androgen-deprived media
ADT: androgen deprivation therapy
AI: androgen-independent
AR: androgen receptor
ARBS: AR binding sites
ARE: androgen response elements
ChIP: chromatin immunoprecipitation
CRPC: castration-recurrent prostate cancer
CSS: charcoal stripped serum
DHT: dihydrotestosterone
ENZ: enzalutamide
FBS: fetal bovine serum
FPKM: Fragments Per Kilobase of transcript per Million
GEO: gene expression omnibus
GFP: green fluorescent protein
Kb: kilobase
mCRPC: castration-recurrent metastatic prostate cancer
PBS: phosphate buffered saline
PC: prostate cancer
qRT-PCR: quantitative reverse transcriptase polymerase chain reaction
SFK: Src-family kinases.
