# Supplementary material for: Src promotes castration-recurrent prostate cancer through androgen receptor-dependent canonical and non-canonical transcriptional signatures
Source: Oncotarget. 2016 Dec 31;8(6):10324–47. doi: 10.18632/oncotarget.14401 (PMC5354662; doi:10.18632/oncotarget.14401)
Supplement: Supplementary file 1 [file oncotarget-08-10324-s001.pdf]

Src promotes castration-recurrent prostate cancer through androgen receptor-dependent canonical and non-canonical transcriptional signatures

SUPPLEMENTARY FIGURES AND TABLES

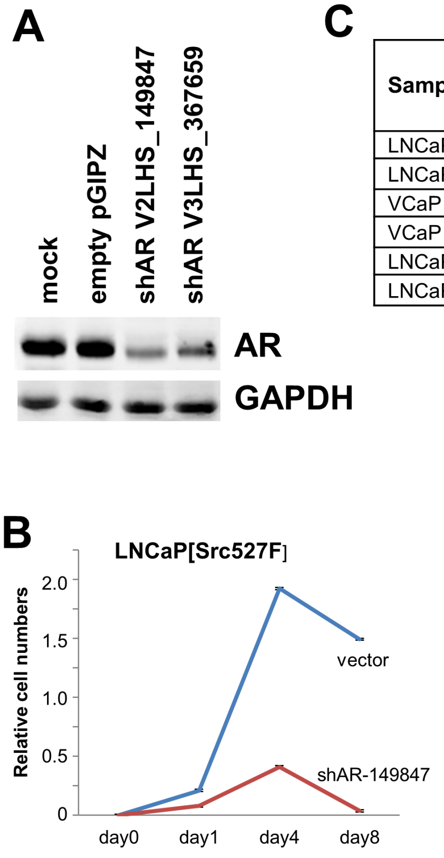

C

Sample ID	Yield (Mb)	Reads/ Sample	% of >=Q30 Bases (PF)	Mean Quality Score (PF)
LNCaP	5,378	105,456,818	98.32	38.8
LNCaP + DHT	10,171	199,430,608	94.62	37.17
VCaP	6,640	130,188,094	97.08	38.27
VCaP + DHT	5,660	110,981,604	96.91	38.26
LNCaP[Src]	10,021	196,491,943	94.66	37.19
LNCaP[Src] + DHT	10,136	198,743,718	94.74	37.22

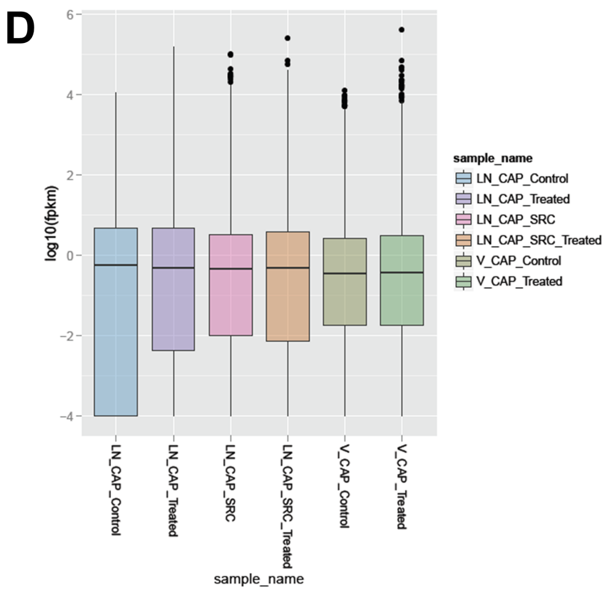

Supplementary Figure 1: Androgen-independent proliferation of LNCaP[Src527F] cells requires AR. A. AR IB of LNCaP[Src^{527F}] cells transduced with empty pGIPZ vector or pGIPZ-shRNA clones specific for AR. GAPDH IB is shown as a protein loading control. **B.** Relative cell numbers of LNCaP[Src^{527F}] cells transduced with empty pGIPZ vector or shAR clone #149847 grown in ADM conditions. **C.** Summary of RNA-seq metrics from ScriptSeq v2 libraries. Mb, megabases. Q, quality value: integer mapping of P (probability that corresponding base is correct). PF, passed filtering. **D.** Boxplot of FPKM (Fragments Per Kilobase of transcript per Million mapped reads) distributions for vehicle- (Control) or DHT-treated (Treated) RNA-seq samples.

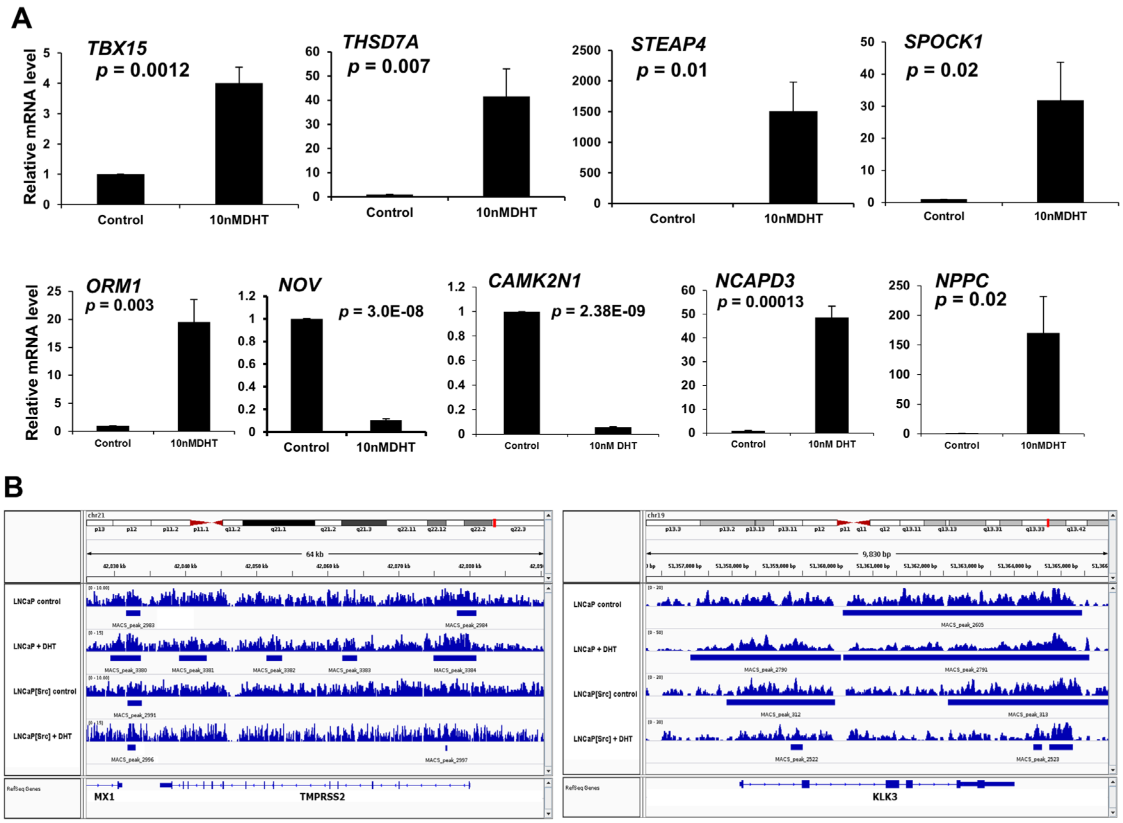

Supplementary Figure 2: A. Validation of DHT-regulated expression of genes identified in Figure 2C by qRT-PCR analysis. Error bars, +/- s.d. of two independent experiments done in triplicate. **B.** MACS peak analyses of the *TMPRSS2* and *KLK3* loci from vehicle- (control) or DHT-treated LNCaP vs. LNCaP[Src^{527F}] cells.

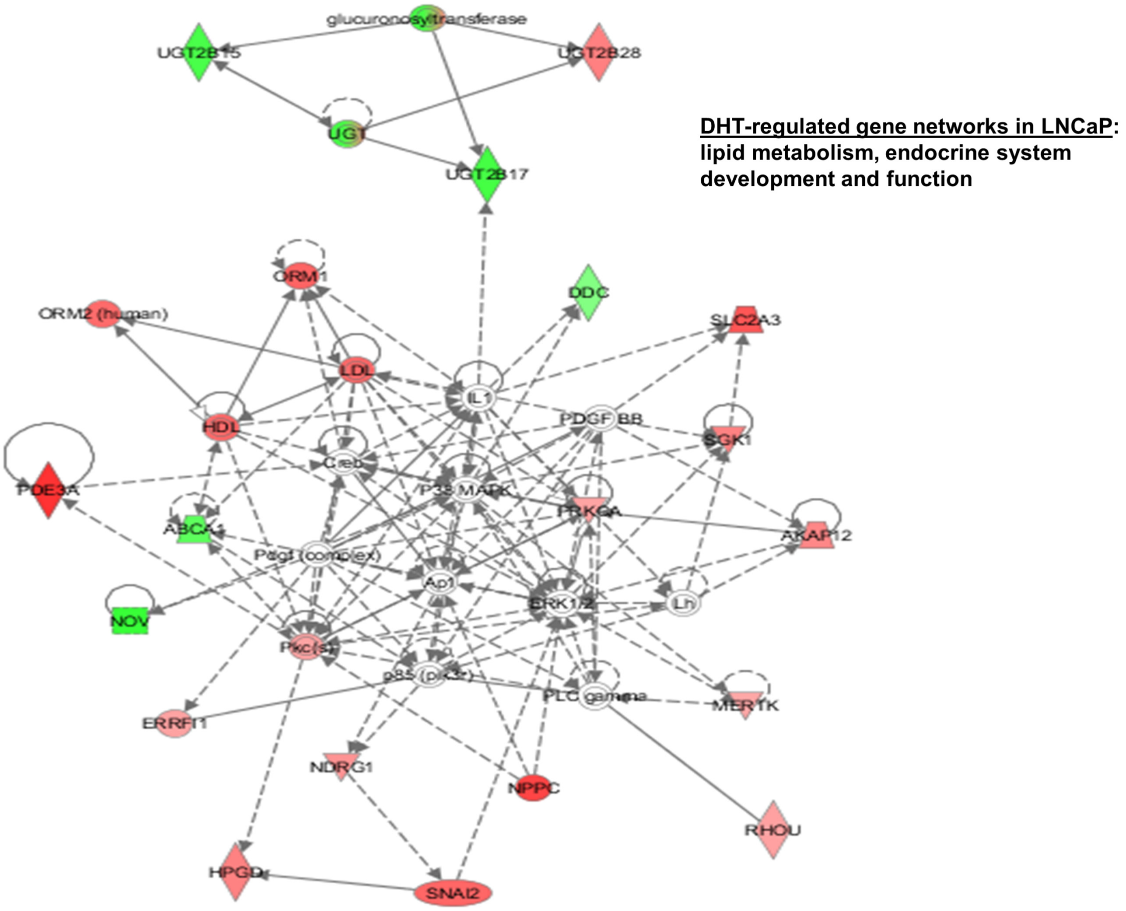

Supplementary Figure 3: Ingenuity Pathway Network analysis of DHT-regulated genes in LNCaP cells, identifying pathways controlling lipid metabolism, and endocrine system development and function. Upregulated genes are in red and downregulated genes are in green.

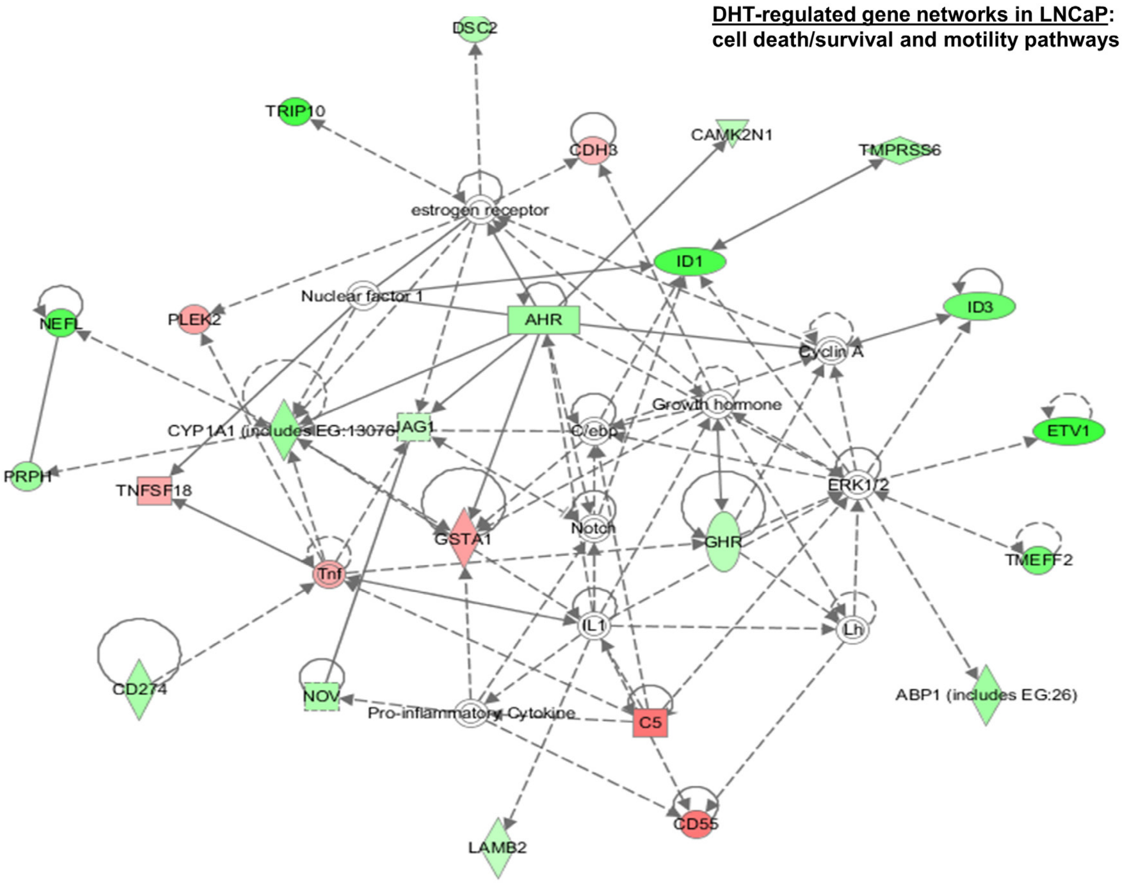

Supplementary Figure 4: Ingenuity Pathway Network analysis of DHT-regulated genes in LNCaP cells, identifying cell death/survival and cell motility pathways. Upregulated genes are in red and downregulated genes are in green.

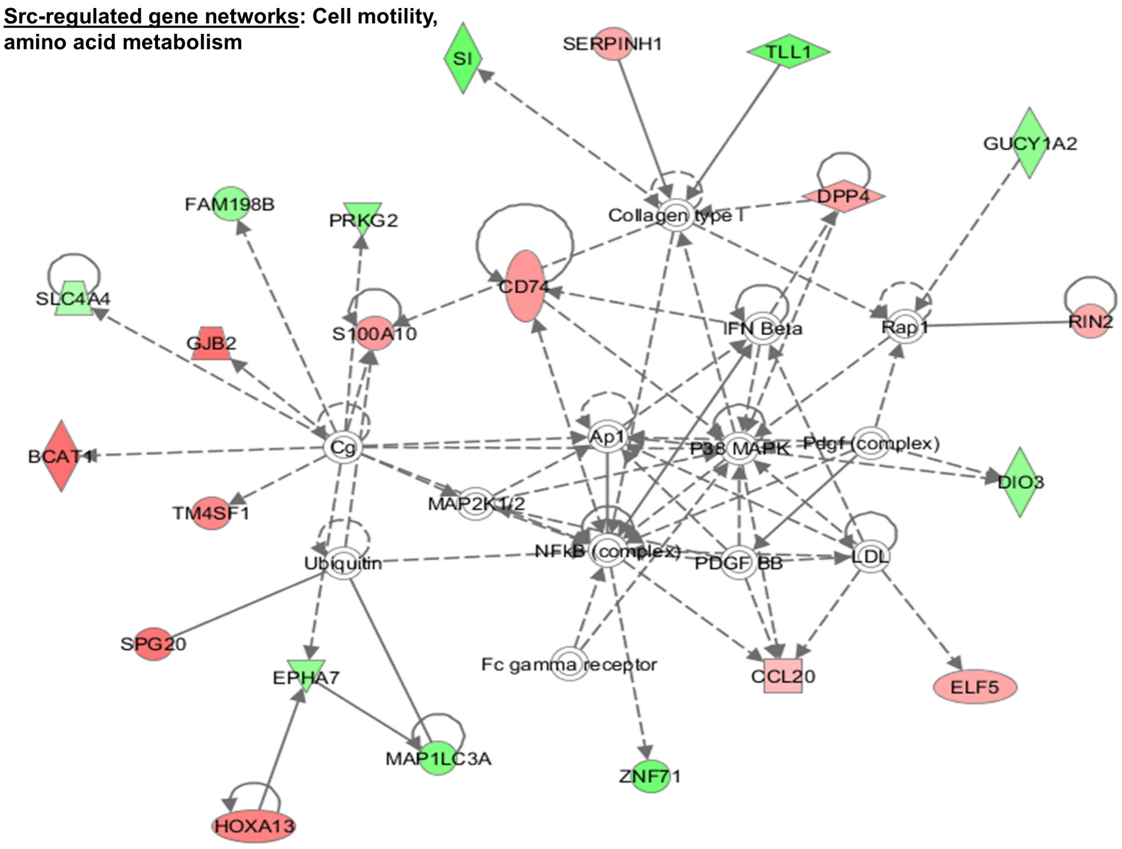

Supplementary Figure 5: Ingenuity Pathway Network analysis of Src-regulated genes in LNCaP cells, identifying cell motility and amino acid metabolism pathways. Upregulated genes are in red and downregulated genes are in green.

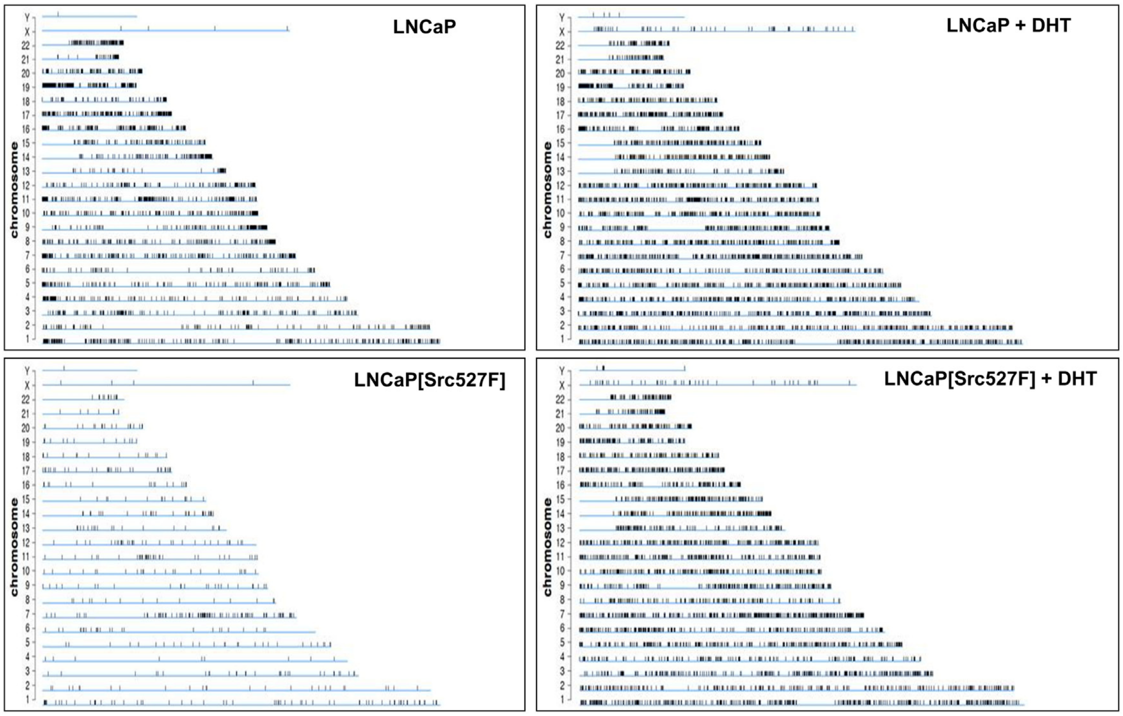

Supplementary Figure 6: ARBS mapping to chromosome marks in LNCaP and LNCaP[Src^{527F}] cells treated with vehicle or DHT.

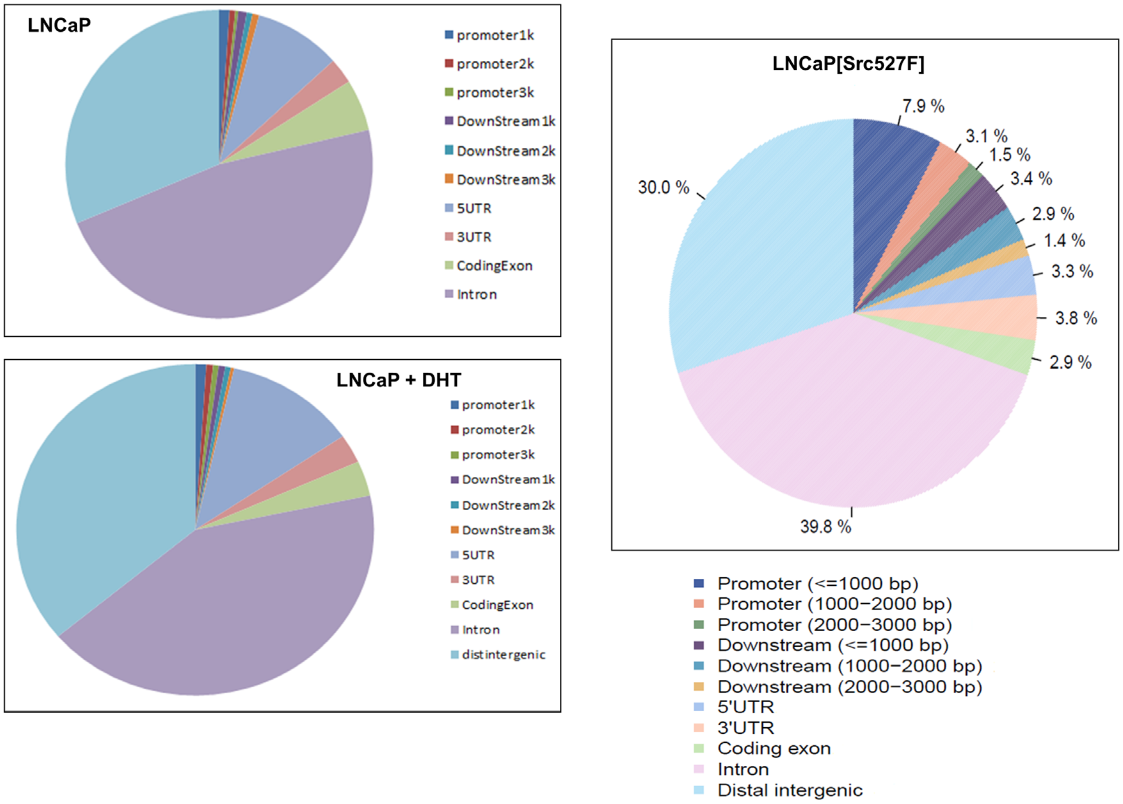

Supplementary Figure 7: Frequency of ARBS mapping to genic and intergenic regions.

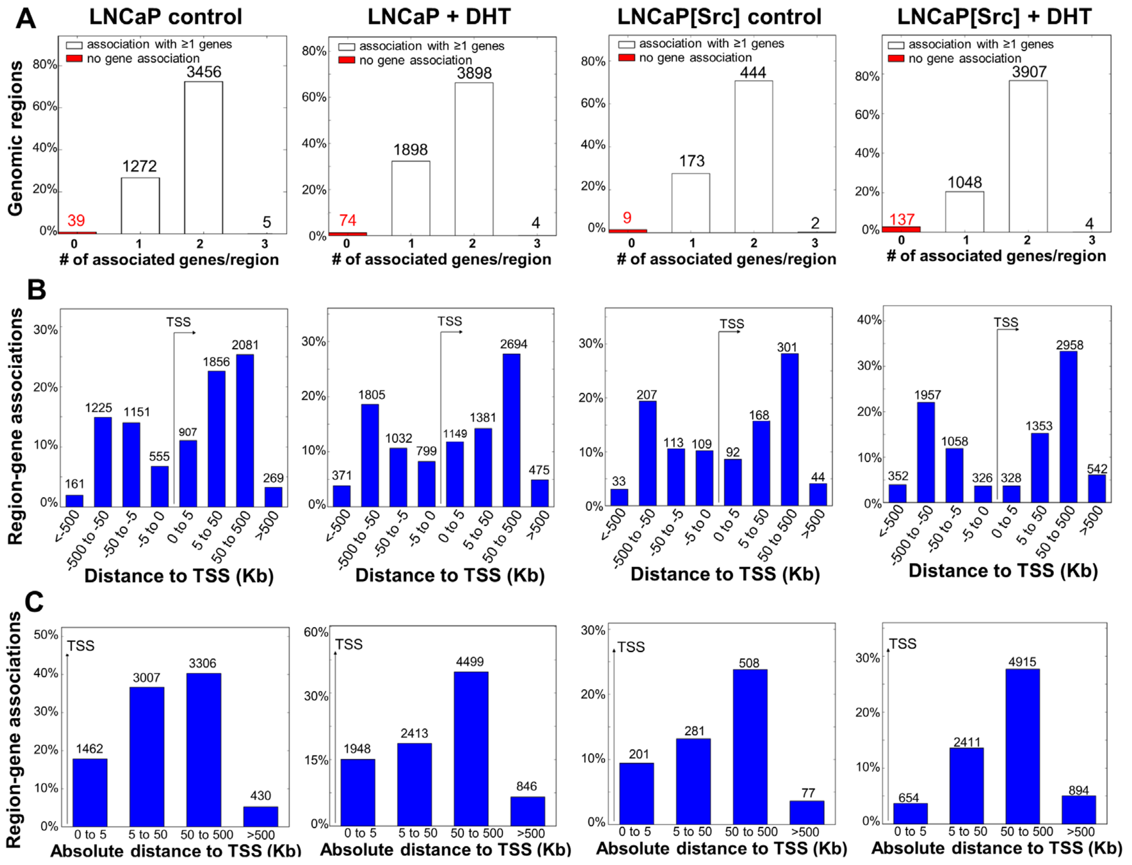

Supplementary Figure 8: GREAT analysis of ARBS relative to gene associations within 50Kb **A.** distance up- or downstream from closest TSS **B.** or absolute distance in Kb from closest TSS **C.** in LNCaP and LNCaP[Src^{527F}] cells treated with vehicle or DHT.

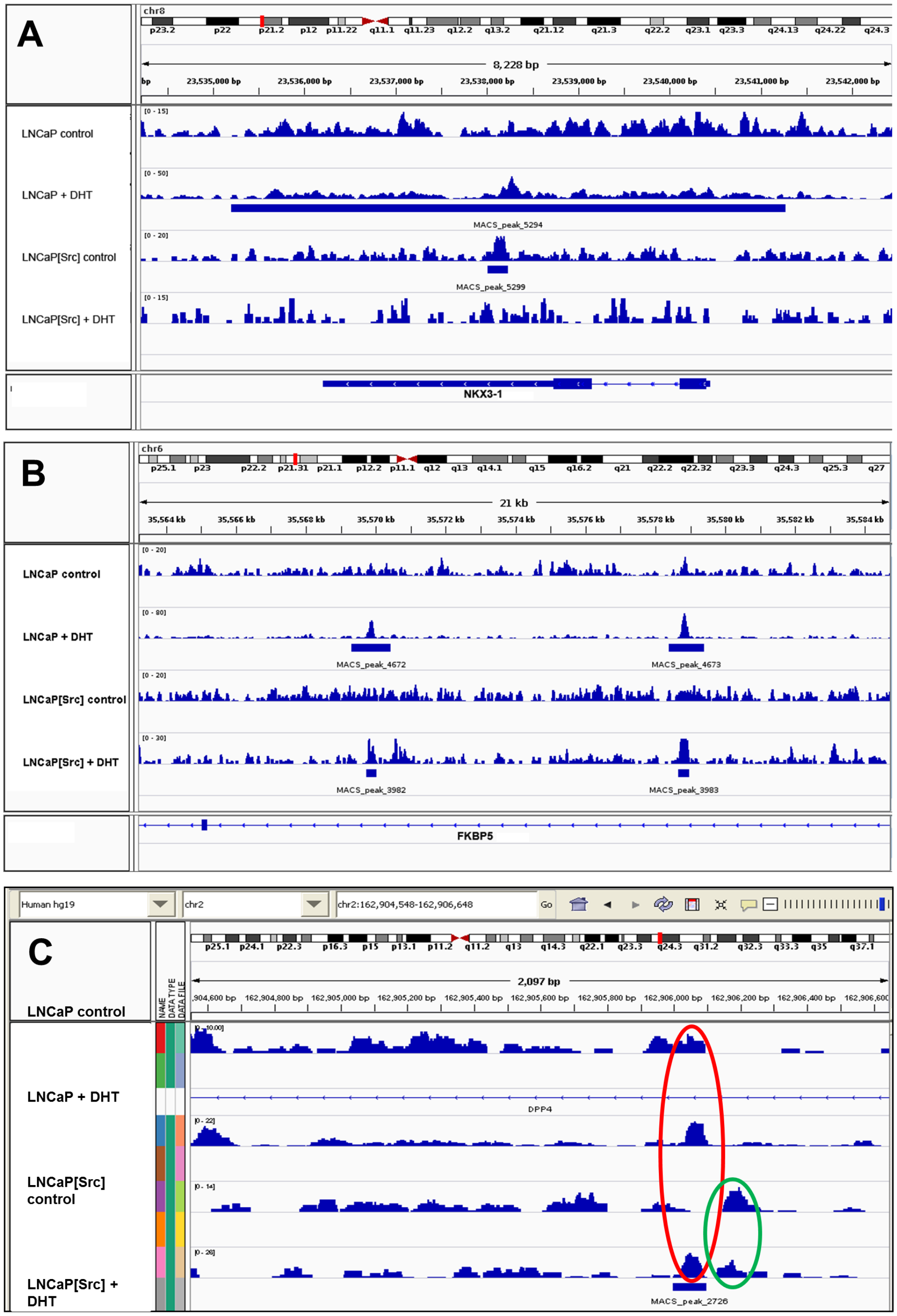

Supplementary Figure 9: Snapshot of ARBS peaks (MACS analysis) for *FKBP5* A. or *NKX3-1* B. enhancer regions comparing peaks from LNCaP and LNCaP[Src^{527F}] cells treated with vehicle (control) or DHT. C. Snapshot of ARBS peaks relative to the *DPP4* enhancer region on chromosome 2. Relative peak scales can be inferred from the bracketed Y-axis values at left. Red circle, ARBS peak #2726 shared by LNCaP-control, LNCaP+DHT and LNCaP[Src^{527F}]+DHT. Green circle, ARBS peak shared by LNCaP[Src^{527F}]-control and LNCaP[Src^{527F}]+DHT.

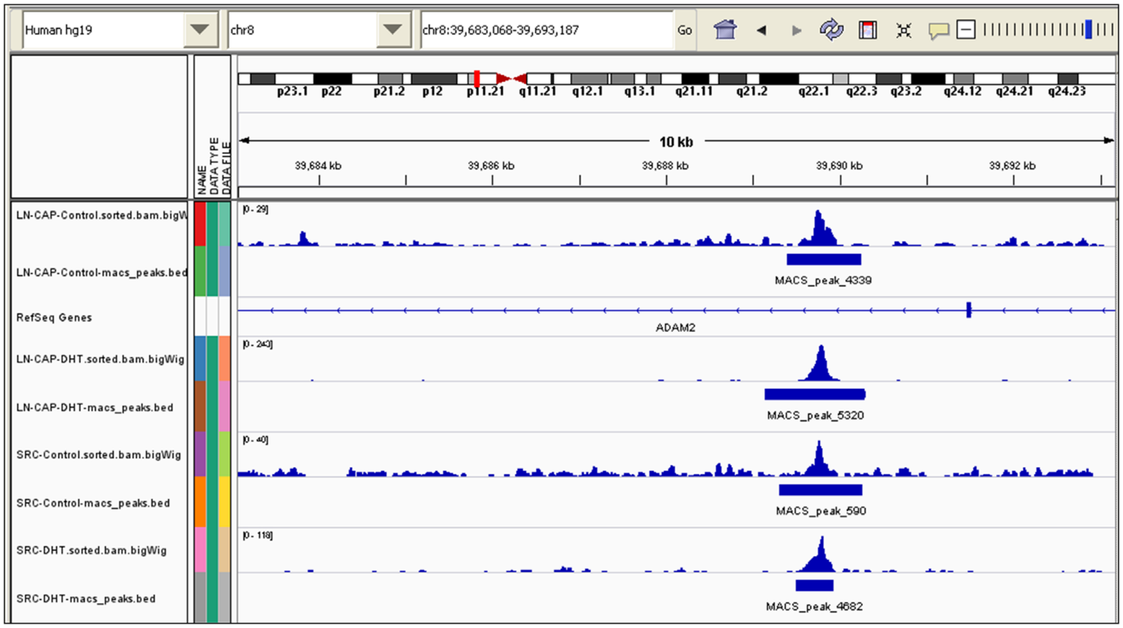

Supplementary Figure 10: Snapshot of ARBS peaks (MACS analysis) relative to the *ADAM2* enhancer region on chromosome 8, showing the overlapping ARBS peak shared by LNCaP and LNCaP[Src^{527F}] cells treated with vehicle (control) or DHT.

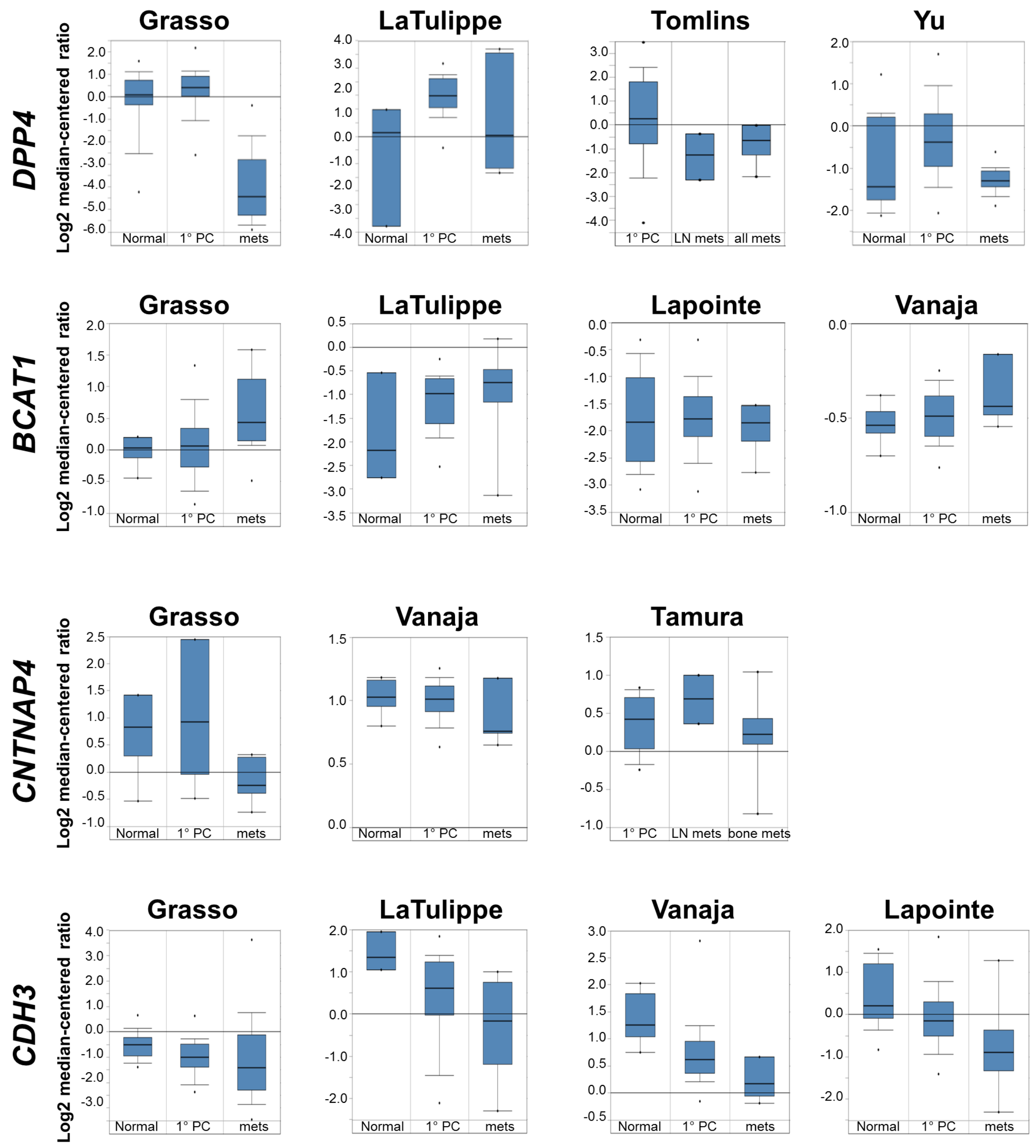

Supplementary Figure 11: Oncomine analyses showing expression trends for *DPP4*, *BCAT1*, *CNTNAP4* and *CDH3* in multiple gene expression studies that compare normal, primary-site (1°) PC and CRPC ("mets") tissues, or in the case of the Tomlins and Tamura studies, 1° PC vs. lymph node (LN) or bone mets, vs. all CRPC lesions.

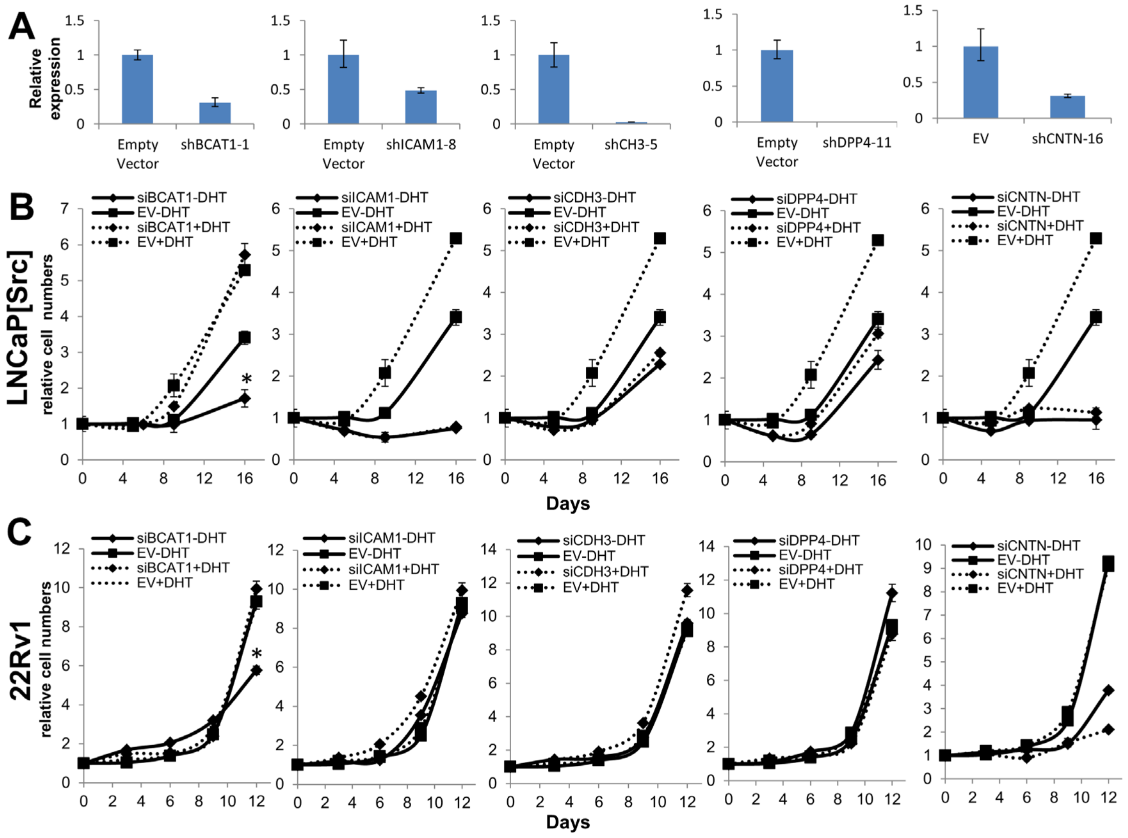

Supplementary Figure 12: Effect of knockdown of members of the 11-gene Src-induced CRPC signature on androgen-independent proliferation. **A.** Relative RNA expression levels of *BCAT1, ICAM1, CDH3, DPP4* or *CNTNAP4* ("CNTN") in LNCaP[Src^{527F}] cells transduced with empty vector (EV) or gene-specific shRNAs, assessed by qRT-PCR. Error bars, mean +/- SEM from triplicates done in 2 independent experiments. Relative numbers of EV- or shRNA-transduced LNCaP[Src^{527F}] **B.** or CWR22Rv1 **C.** cells (assayed as described in Materials and Methods) grown in the absence (-) or presence (+) of 1 nM DHT. Error bars, mean +/- SEM from triplicates done in 2 independent experiments. *, P<0.01.

A

LuCaP35.1

(PC1 vs. CR1) vs. (PC2 vs. CR2)			All PC vs. All CR
C11orf92	IFI6*	OAS1*	DDX58*
CACNB4*	IFIH1*	OAS3*	IFI27*
CD177	IFIT1*	PTPN13*	IFI44*
CLGN	IFIT2*	RSAD2*	IFI44L*
CMPK2*	IFIT3*	SAMD9*	IFI6*
DDX58*	IFITM3*	SLC26A2*	IFIT1*
GAGE2A	IGF2BP1	SP110*	IGF2BP1
GDF15*	ISG15*	SPON2*	MX1*
HLA-A*	MATN2		NPY
IFI27*	MX1*		PTPN13*
IFI44*	MX2*		SAMD9*
IFI44L*	NPY		SLC26A2*

* Interferon-inducible genes (http://www.interferome.org)

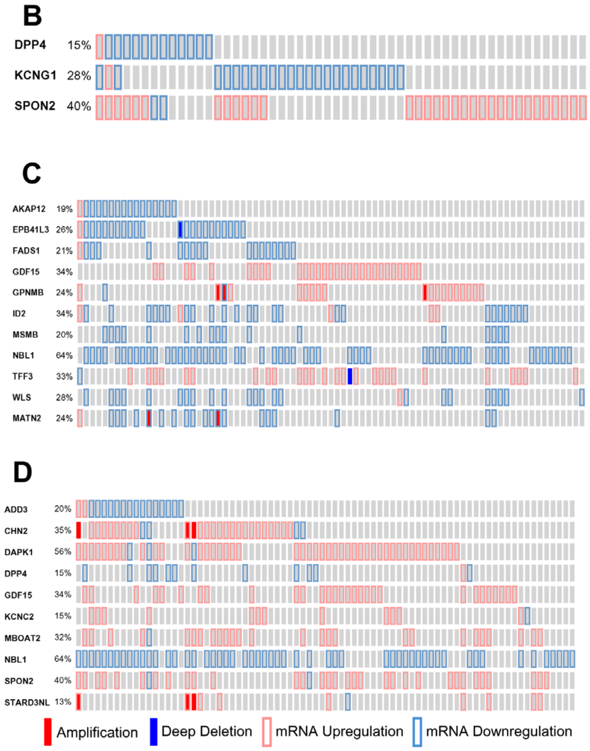

Supplementary Figure 13: A. Differentially-expressed genes (threshold of $\log_2^{\geq 3}$) between AD (PC) or CRPC LuCaP35.1 xenografts. *, identification as interferon-inducible genes (http://www.interferome.org). Differential expression trends of the CRPC-associated gene sets identified in Figs. 7C, D and E that compare LuCaP35.1 CR-transcriptomes with those induced by Src527F in LNCaP **B.**, CRPC clinical lesions **C.**, and CRPC-associated ARBS **D.** The Oncomine gene set used for comparison here is that of Taylor et al. [82] for primary PC.

Supplementary Table 1: List of qRT-PCR primer sets and amplicon sizes

Gene Name	Forward Primer	Reverse Primer	Amplicon Size (bp)
TBX15	ATGTGATTCGCAAAGACTTCAGC	GATAGGCCGTAACTGTGGTGA	117
THSD7A	TCAGTGTCAGCCCGTGATTTC	GATGCACGCTATCTCCCTCA	94
STEAP4	GGCTTTGGGAATACTTGGGTT	TGGACAAATCGGAACTCTCTCC	102
SPOCK1	CCCAACCACGGCAATTTCCTA	ATCGTCTCGAAAGCGGTTCC	93
NOV	AACTGCATTGAACAGACCACA	ATTGACGGTTCCTATTGGTGAC	91
ORM1	ACACCACCTACCTGAATGTCC	GTGAGCGAAATGCTCTTGGC	77
NCAPD3	GTGCCAAGGTGGTAGATAAATCA	AACATAGCGTATTCCCCACAAG	97
TM4SF1	TGTGGCAAACGATGTGCGA	TGACACAGTAGCCAGATCCTG	76
DPP4	TACAAAAGTGACATGCCTCAGTT	TGTGTAGAGTATAGAGGGGCAGA	134
SPG20	ATGGAGCAAGAGCCACAAAAT	TCTGAGCAGGTGTCCTATTCC	165
GJB2	TCGCATTATGATCCTCGTTGTG	GGGGAAGTAGTGATCGTAGCAC	118
ICAM1	ATGCCCAGACATCTGTGTCC	GGGGTCTCTATGCCCAACAA	112
BCAT1	GAGCCTGGAAAGGTGGAACTG	GCTGACACCCATTATCTACTGCT	96
CNTNAP4	AGCAGTCATGGTCCTGGATTT	TCTGCAACCACTGGTATTTGTT	94
CDH3	TGGAGATCCTTGATGCCAATGA	GCGTCCAGATCAGTGACCG	121
AR	CCTGGCTTCCGCAACTTACAC	GGACTTGTGCATGCGGTACTCA	168
PSA	TGTGTGCTGGACGCTGGA	CACTGCCCCATGACGTGAT	105
TMPRSS2	GGACAGTGTGCACCTCAAAGAC	TCCCACGAGGAAGGTCCC	71
TOP2B	TTTGAATGGGGCTGCGGCCCC	TTCTCCATTTCCTCATCTGTAA	138
CDX2	TTAGCCGGGCGTGGTGGCACATGCC	CCGTCGAAACGCAGTTTCACTCTTGTTGTG	153

Supplementary Table 2: Differentially expressed gene lists (threshold of $log_2^{\geq 3}$) shared by comparing various PC cell lines +/-DHT treatment. Genes also identified as regulated by both DHT and Src (*, Figure 4B), as DHT regulated genes affected by Src (‡, Figure 4D), or as in the 11-gene Src-regulated CRPC signature (§, Figure 5D) are shown in bold.

See Supplementary File 1

Supplementary Table 3: Genes differentially expressed at a threshold of $log_2^{\geq 3}$over background under various cell and DHT treatment condition comparisons.

See Supplementary File 2

Supplementary Table 4: Genes differentially expressed at a threshold of $log_2^{\geq 3}$over background under various cell and DHT treatment condition comparisons.

See Supplementary File 3

Supplementary Table 5: Genes differentially expressed at a threshold of $log_2^{\geq 3}$over background under various cell and DHT treatment condition comparisons.

See Supplementary File 4

Supplementary Table 6: Genes differentially expressed at a threshold of $log_2^{\geq 3}$over background under various cell and DHT treatment condition comparisons.

See Supplementary File 5

Supplementary Table 7: Genes differentially expressed at a threshold of $log_2^{\geq 3}$over background under various cell and DHT treatment condition comparisons.

See Supplementary File 6

Supplementary Table 8: Genes differentially expressed at a threshold of $\log_2^{\geq 3}$over background under various cell and DHT treatment condition comparisons.

See Supplementary File 7

Supplementary Table 9: Genes differentially expressed at a threshold of $\log_2^{\geq 3}$over background under various cell and DHT treatment condition comparisons.

See Supplementary File 8

Supplementary Table 10: Genes differentially expressed at a threshold of $\log_2^{\geq 3}$over background under various cell and DHT treatment condition comparisons.

See Supplementary File 9

Supplementary Table 11: Genes differentially expressed in VCaP cells treated with vehicle (control) vs. DHT (10 nM)

See Supplementary File 10

Supplementary Table 12: Shared genes regulated by Src in LNCaP cells (+/- DHT) vs. DHT-treated LNCaP-C4-2 cells

See Supplementary File 11

Supplementary Table 13: Shared genes regulated by Src in LNCaP cells vs. vehicle-treated (control) LNCaP-C4-2 cells

See Supplementary File 12
