# Supplementary material for: Src promotes castration-recurrent prostate cancer through androgen receptor-dependent canonical and non-canonical transcriptional signatures
Source: Oncotarget. 2016 Dec 31;8(6):10324–47. doi: 10.18632/oncotarget.14401 (PMC5354662; doi:10.18632/oncotarget.14401)
Supplement: Supplementary file 2 [file oncotarget-08-10324-s002.docx]

**Table S2A**

| **Genes Common to:** | | |
| --- | --- | --- |
| **(LNCaP vs. LNCaP[Src]) & (C4-2 vs. C4-2+DHT)** | **(LNCaP+DHT vs. LNCaP[Src]) &**  **(C4-2 vs. C4-2+DHT)** | |
| ADAM7 | ADAM7 | **MYBPC1 ‡** |
| ARHGEF38 | ARHGEF38 | **NDRG1 ‡** |
| **DPP4 §** | BMPR1B | **NKX3-1 ‡** |
| DSC2 | CYP2U1 | NPPC |
| **ELF5 §** | **DHCR24 ‡** | PAGE1 |
| GUCY1A2 | **DPP4 §** | PAK1IP1 |
| ID3 | **ELF5 §** | PNLIPRP3 |
| **JAG1 §** | ELL2 | PRSS1 |
| PAGE1 | ENDOD1 | PSMD8 |
| PRSS1 | ERRFI1 | PTPRM |
| RIN2 | FADS2 | RHOU |
| SLC45A3 | FAM105A | RIN2 |
| **SNORA24 *** | GUCY1B3 | RND3 |
| **SNORD104 *** | HOMER2 | **SGK1 ‡** |
| **SNORD1C *** | HPGD | SLC45A3 |
| **TM4SF1 §** | ID3 | **SNAI2 ‡** |
| TTC29 | **JAG1 §** | STK17B |
|  | LCP1 | STK39 |
|  | LONRF1 | TBC1D4 |
|  | LPAR3 | **TM4SF1 §** |
|  | MAF | WIPI1 |
|  | MAP7D1 |  |

*** identified in Fig. 4B**

**‡ identified in Fig. 4D**

**§ identified in Fig. 5D**

**Table S2B**

| **Genes common to:** | | | | | | | | |
| --- | --- | --- | --- | --- | --- | --- | --- | --- |
| **(LNCaP vs. LNCaP[Src]) &**  **(LNCaP vs. C4-2)** | | | **(LNCaP+DHT vs. LNCaP[Src]) & (LNCaP vs. C4-2)** | | | **LNCaP[Src] &**  **C4-2** | | |
| ABP1 | GNAQ | SLFN12 | ABP1 | GNAQ | SLFN12 | ADAM7 | GEMC1 | SPON2 |
| ADAM7 | GPR108 | SLITRK3 | ADAM7 | GPR108 | SLITRK3 | ADAMTSL3 | GHR | TLL1 |
| ADAMTSL3 | GPR158 | SMOC2 | ADAMTSL3 | GPR158 | SMOC2 | ADRA2A | **GJB2 §** | **TM4SF1 §** |
| ADRA2A | GRIK1 | **SNORA24 *** | ADRA2A | GRIK1 | **SNORA24 *** | AHR | GNAQ | TMEFF2 |
| AHR | GSTA1 | **SNORD104 *** | AHR | GSTA1 | **SNORD104 *** | ALDH1L1 | GPR108 | TOX3 |
| ALDH1L1 | HIST1H2BG | **SPG20 §** | ALDH1L1 | HIST1H2BG | **SPG20 §** | ALDH1L2 | GPR158 | TRIP10 |
| ALDH1L2 | HOXA13 | SPON2 | ALDH1L2 | HOXA13 | SPON2 | ARHGEF38 | GSTA1 | TXLNB |
| ANKRD30A | ICAM1 § | **STAC *** | ANKRD30A | ICAM1 § | STAC | **BCAT1 §** | **ICAM1 §** | USP9Y |
| ARHGEF38 | ICAM5 | STOM | ARHGEF38 | ICAM5 | STOM | C10orf58 | ID1 | VCY |
| **BCAT1 §** | ID1 | **SYT4 *** | **BCAT1 §** | ID1 | **SYT4 *** | C5orf42 | ID3 | ZC4H2 |
| **BCHE *** | ID3 | TLL1 | **BCHE *** | ID3 | TLL1 | CACNA1H | ITPRIPL2 | ZFP28 |
| C10orf58 | ITPRIPL2 | **TM4SF1 §** | C10orf58 | ITPRIPL2 | **TM4SF1 §** | CARTPT | JAG1 § | ZFY |
| C11orf92 | **JAG1 §** | TMC1 | C11orf92 | JAG1 § | TMC1 | CBLN2 | KCNH8 | ZIK1 |
| C5 | KCNG1 | TMEFF2 | C5 | KCNG1 | TMEFF2 | CCDC85A | KCNJ3 | ZNF20 |
| C5orf38 | KCNH8 | TMEM121 | C5orf38 | KCNH8 | TMEM121 | **CD274 §** | KIAA2022 | ZNF208 |
| C5orf42 | KCNJ3 | TMEM144 | C5orf42 | KCNJ3 | TMEM144 | CD55 | KLHL1 | ZNF256 |
| CACNA1H | KIAA2022 | TMPRSS6 | CACNA1H | KIAA2022 | TMPRSS6 | **CDH3 §** | LOC100128252 | ZNF383 |
| **CAMK2N1 *** | KLHL1 | TOX3 | **CAMK2N1 *** | KLHL1 | TOX3 | CDR1 | MAP1LC3A | ZNF433 |
| **CARTPT ‡** | LAMB2 | TPTE | CARTPT | LAMB2 | TPTE | CLIP4 | NAP1L2 | ZNF442 |
| CBLN2 | LHX6 | TRIM47 | CBLN2 | LHX6 | TRIM47 | **CNTNAP4 §** | NEFL | ZNF470 |
| CCDC85A | LOC100128252 | TRIP10 | CCDC85A | LOC100128252 | TRIP10 | COX7B2 | PABPC5 | ZNF558 |
| CD177 | LOC348021 | TTC29 | CD177 | LOC348021 | TTC29 | CSRNP3 | PAGE1 | ZNF560 |
| **CD274 §** | LONRF3 | TUBB4 | **CD274 §** | LONRF3 | TUBB4 | CYP1A1 | PCDH11X | ZNF563 |
| CD55 | MAGEA1 | TXLNB | CD55 | MAGEA1 | TXLNB | CYorf15A | PCDHB2 | ZNF570 |
| CD74 | MAP1LC3A | USP9Y | CD74 | MAP1LC3A | USP9Y | CYorf15B | PCDHB5 | ZNF585A |
| **CDH3 §** | MIMT1 | VCY | **CDH3 §** | MIMT1 | VCY | DDX3Y | PGCP | ZNF585B |
| CDR1 | MLANA | ZBBX | CDR1 | MLANA | ZBBX | DEFB132 | PRSS1 | ZNF625 |
| CLIP4 | NAP1L2 | ZC4H2 | CLIP4 | NAP1L2 | ZC4H2 | DNAJC15 | ROBO1 | ZNF667 |
| CNBD1 | NAP1L3 | ZFP28 | CNBD1 | NAP1L3 | ZFP28 | **DPP4 §** | SEMA6A | ZNF676 |
| **CNTNAP4 §** | NCRNA00221 | ZFY | **CNTNAP4 §** | NCRNA00221 | ZFY | EIF1AY | SLC22A3 | ZNF71 |
| COX7B2 | NEFL | ZIK1 | COX7B2 | NEFL | ZIK1 | **ELF5 §** | SLC25A43 | ZNF737 |
| CSRNP3 | **NOV *** | ZNF20 | CSRNP3 | **NOV *** | ZNF20 | EPHA7 | SLC45A3 | ZNF77 |
| CYorf15A | NUP93 | ZNF208 | CYorf15A | NUP93 | ZNF208 | ETV1 | SLC4A4 | ZNF844 |
| CYorf15B | **OPRK1 *** | ZNF256 | CYorf15B | **OPRK1 *** | ZNF256 | EYA2 | SLFN12 | ZNF90 |
| CYP1A1 | OR51E1 | ZNF383 | CYP1A1 | OR51E1 | ZNF383 | GALC | **SPG20 §** | ZNF91 |
| DDIT4L | PABPC5 | ZNF433 | DDIT4L | PABPC5 | ZNF433 |  |  | ZSCAN18 |
| DDX3Y | PAGE1 | ZNF442 | DDX3Y | PAGE1 | ZNF442 |  | | |
| DEFB132 | PAK1 | ZNF470 | DEFB132 | PAK1 | ZNF470 |  |  |  |
| DIO3 | PCDH11X | ZNF491 | DIO3 | PCDH11X | ZNF491 |  |  |  |
| DNAJC15 | PCDHB11 | ZNF558 | DNAJC15 | PCDHB11 | ZNF558 |  |  |  |
| **DPP4 §** | PCDHB2 | ZNF560 | **DPP4 §** | PCDHB2 | ZNF560 |  |  |  |
| DPY19L2P2 | PCDHB5 | ZNF563 | DPY19L2P2 | PCDHB5 | ZNF563 |  |  |  |
| DSC2 | PGCP | ZNF570 | DSC2 | PGCP | ZNF570 |  |  |  |
| EFNB3 | PLEK2 | ZNF585A | EFNB3 | PLEK2 | ZNF585A |  |  |  |
| EIF1AY | PRAC | ZNF585B | EIF1AY | PRAC | ZNF585B |  |  |  |
| **ELF5 §** | PRKG2 | ZNF619 | **ELF5 §** | PRKG2 | ZNF619 |  |  |  |
| EPHA7 | PRPH | ZNF625 | EPHA7 | PRPH | ZNF625 |  |  |  |
| ETV1 | PRSS1 | ZNF667 | ETV1 | PRSS1 | ZNF667 |  |  |  |
| EYA2 | ROBO1 | ZNF676 | EYA2 | ROBO1 | ZNF676 |  |  |  |
| FAM186A | S100A10 | ZNF709 | FAM186A | S100A10 | ZNF709 |  |  |  |
| FAM198B | SEMA6A | ZNF71 | FAM198B | SEMA6A | ZNF71 |  |  |  |
| FAM20C | SERPINH1 | ZNF736 | FAM20C | SERPINH1 | ZNF736 |  |  |  |
| FBXL7 | **SI *** | ZNF737 | FBXL7 | **SI *** | ZNF737 |  |  |  |
| GALC | SLC22A3 | ZNF77 | GALC | SLC22A3 | ZNF77 |  |  |  |
| GCG | SLC25A43 | ZNF844 | GCG | SLC25A43 | ZNF844 |  |  |  |
| GEMC1 | SLC45A3 | ZNF90 | GEMC1 | SLC45A3 | ZNF90 |  |  |  |
| GHR | SLC4A10 | ZNF91 | GHR | SLC4A10 | ZNF91 |  |  |  |
| GJB2 § | SLC4A4 | ZSCAN18 | **GJB2 §** | SLC4A4 | ZSCAN18 |  |  |  |
|  |  |  |  |  |  |  |  |  |
